# Menu labeling, calories, and nutrient density: Evidence from chain restaurants

**DOI:** 10.1371/journal.pone.0232656

**Published:** 2020-05-07

**Authors:** Daniel E. Ho, Oluchi Mbonu, Anne McDonough, Rebecca Pottash

**Affiliations:** 1 William Benjamin Scott and Luna M. Scott Professor of Law, Professor of Political Science, Senior Fellow, Stanford Institute for Economic and Policy Research, Stanford University, Stanford, CA, United States of America; 2 Ph.D. Student, Harvard University, Cambridge, MA, United States of America; 3 Research Fellow, Stanford Law School, Stanford, CA, United States of America; 4 J.D., Stanford Law School, Stanford, CA, United States of America; University of Florida, UNITED STATES

## Abstract

The Food and Drug Administration’s menu labeling rule requires chain restaurants to prominently display calories, while leaving other nutritional information (e.g., fat, sodium, sugar) to the request of consumers. We use rich micronutrient data from 257 large chain brands and 24,076 menu items to examine whether calories are correlated with widely used “nutrient profile” scores that measure healthfulness based on nutrient density. We show that calories are indeed statistically significant predictors of nutrient density. However, as a substantive matter, the correlation is highly attenuated (partial R^2^ < 0.01). Our findings (a) suggest that the promise of calorie labeling to improve nutrient intake quality at restaurants is limited and (b) clarify the basis for transparency of nutrient composition beyond calories to promote healthy menu choices.

## I. Introduction

Pursuant to the Affordable Care Act (ACA), the Food and Drug Administration (FDA) mandated menu labeling at chain restaurants with over 20 locations starting in May of 2018. While an earlier legislative proposal would have required menu labeling of five nutrient attributes (saturated fat, trans fat, carbohydrates, sodium, and calories) [1], the ACA requires chains to post solely calories on menus [2]. Disclosure of other nutrient information (e.g., sodium, sugar, dietary fiber) is left to the request of the consumer, which functionally limits disclosure to calories, as only 0.1% of patrons access nutrition information before purchase [3].

The regulatory choice to prioritize calories at the exclusion of other nutrients raises considerable tension in food law and policy, a field relying critically on disclosure [4]. On the one hand, simplifying information to a single attribute can increase salience to consumers [5–7]. Faced with cognitive overload, consumers may be better able to make comparisons on a single attribute [8]. On the other hand, the U.S. Dietary Guidelines are more complex, urging consumers to “choose a variety of nutrient-dense foods across and within all food groups” to “meet nutrient needs within calorie limits” [9]. Correspondingly, FDA requires “Nutrition Facts” labels for packaged food to disclose calories, fat, saturated fat, trans fat, carbohydrates, cholesterol, sodium, dietary fiber, sugar, vitamin A, vitamin C, calcium, and iron (21 C.F.R. § 101.9). Similarly, the Institute of Medicine (IOM) endorsed increased nutrient disclosure in the retail food environment when it concluded that at least four nutrients (calories, saturated fat, trans fat, sodium) should be disclosed on front-of-package labels [10]. Local jurisdictions, like Philadelphia and King County (WA), have required restaurants to list other nutrient attributes in addition to calories on menus [11,12]. Singling out calories may distort the balance of nutritional intake.

As a matter of nutritional science, many defend the focus on calories on the grounds that calories are the single most important piece of information for consumers, particularly in light of the public health challenges of obesity [6,13]. By incentivizing consumers to lower portion sizes, Marion Nestle argues, the regulation may help lower obesity rates [14]. Others dispute the preoccupation with calorie counts [15]. Poor health may stem not only from high-calorie foods, but from overconsuming low-nutrient and underconsuming of high-nutrient foods [16–21]. Notes Walter Willett, “The quality of our calories… has an important effect on whether we gain or lose body fat” [22]. Dariush Mozaffarian, who along with David Ludwig advocates a focus on food, not discrete nutrients [23], concludes that “[j]ust counting calories won’t matter much unless you look at the kinds of calories you’re eating” [22]. From that perspective, the regulatory choice to limit disclosure to calories may echo George Loewenstein’s provocative argument that the food industry embraced calorie labeling as “the lesser of evils” to ward off more substantive and effective reform [24].

Regardless of the exact importance of calories as a matter of nutritional science, understanding the relationship between calories and nutritional quality is important as a policy matter in light of the ACA compromise. Even those who view calories as the most important attribute acknowledge the importance of nutritional *quality* [6,25,26]. The Institute of Medicine, for instance, blames obesity on “high-calorie and *low-nutrient*” foods [27]. As Adam Drewnowski puts it, “If we tell consumers to count calories, we must also tell them to make each calorie count more” [18]. Yet it is unclear whether restaurant foods that are lower calorie–or those that are reformulated to be lower calorie–are necessarily associated with improved nutrient content. Some conjecture that calories, even if not the most important nutritional factor, signal the overall healthfulness of an item. FDA’s Obesity Working Group notably found that consumer support for calorie labeling stemmed from the belief “that calories could be a signal for the level of other macronutrients” [6]. Based on a randomized controlled trial of calorie claims and disclosure, researchers inferred that a “low calorie claim creates a positive generalization about the product, or a ‘health halo’” [28]. Similarly, economists have posited that calorie labeling will “rais[e] awareness of healthy eating” [29]. Joanne Slavin argues that the mechanism of “reduc[ing] calories [will, in turn] increase nutrient density” [30]. And in announcing its support for calorie labeling, the American Heart Association noted that calorie labeling “may drive the restaurant to reformulate offerings with healthier ingredients” [31].

How strong is the association between calories and healthfulness in restaurant food items? Could the association mitigate, if not resolve, the seeming tension in food policy? And if not, what tradeoffs must be recognized when disclosure focuses on calorie labeling in lieu of more comprehensive labeling? While much research has focused on whether menu labeling reduces the average calorie count of purchases in restaurants [29,31–38] and whether calorie labeling causes restaurants to lower caloric content [25,39–42] we are aware of no study examining whether calories actually signal the overall healthfulness of covered food items under FDA’s labeling rule.

We contribute to the understanding of menu labeling by studying rich micronutrient data from 257 large chain brands for 24,076 items. These chains cover over 168,579 physical store locations, comprising close to one third of estimated brands in the U.S. [43]. Our basic approach is to apply leading nutrient profile scores–designed by others to measure overall healthfulness of food based on eleven nutrient inputs–to this chain restaurant data. The FDA itself has contemplated deploying such nutrient density indices to indicate healthy choices, but has acknowledged that “there is a need for research to determine the best way(s) to present nutrient content information to consumers so that they will make healthier choices when eating food away from home” [6]. These nutrient profiling methods have been proposed, promoted, and deployed across a wide range of jurisdictions to classify healthy / nutritious foods for regulatory purposes, such as setting school food standards and food tax and subsidy programs [44–51]. Such scores are also widely used to rate the nutrient quality of foods (e.g., by the Rudd Institute [52,53], GoodGuide [54], and nutrition scientists [55,56]).

Our paper proceeds as follows. Section II discusses data and methods. Section III provides results and Section IV discusses limitations. Section V concludes with implications.

## II. Data and methods

### A. Nutrition data

We use data from Nutritionix, a commercial online platform that aggregates nutrition data from brands, primarily for personalized health applications. Nutritionix collects information from brands about the nutritional content of their menu items, which they are required, by the FDA under the ACA, to provide upon consumers’ request. In other words, what brands report to Nutritionix is the same information consumers are entitled to under the ACA if they go to the counter. A key advantage of the Nutritionix dataset is its size. Prior studies on the nutritional content of retail food offerings have often used data from only a small number of items, brands, or jurisdictions [3,29,40,57]. Studies that have drawn on larger, national samples have often used MenuStat, which aggregates nutrition information from at most 200 brands depending on the year [58]. For example, Bleich et al. and Wolfson et al. study trends in calories and nutrients before the federal labeling requirement went into effect at 66 restaurant chains included in the MenuStat database [59,60]. Schoffman et al. examine differences across restaurant types in the calorie contents of menu items using data from 150 chains available on MenuStat in 2014 [61]. In contrast, to our knowledge, Nutritionix provides the most comprehensive coverage of retail restaurant nutrition information, with data from over 700 brands, primarily chains, that have more than 270,000 retail locations across the United States. Included in the Nutritionix data are the 100 largest restaurant brands in the country as measured by sales [62]. (Other than serving as the source of data, Nutritionix had no involvement with this research study.) The dataset is organized at the brand-item level, where each observation in the data consists of a unique menu item offered at a brand as of January 2018. Regardless of number of locations, items appear only once in our dataset. For each item, we have brand information (name, number of locations) and item information (name, calories, nutrient attributes (sodium, protein, dietary fiber, saturated fat, cholesterol, sugars, calcium, iron, vitamin A, vitamin C), serving size). While many brands are part of the Nutritionix database, many report only a subset of nutrient attributes, making the calculation of conventional nutrient profile scores impossible. We hence include brands only if at least one menu item had sufficient nutrient information to calculate a nutrient profile score. 302 brands meet this criterion (see SI Section E for a listing in S1 File). A substantially equivalent dataset can be secured by using the application programming interface offered by Nutritionix. Table 1 provides summary statistics about the sample, with nutrients ordered by missingness in the excluded brands. Nutrients that are most frequently missing are calcium, iron, vitamin A, and vitamin C, as the menu labeling rule does not require these nutrients to be available even upon request. Table 1 also shows that included and excluded brands are comparable on observable nutrient components, so there are no obvious substantive mechanisms suggesting selection bias in the included brands. (While the sugar content of items at included brands is significantly higher than at excluded brands, our results are identical if we use a trimmed sample that is balanced on all nutrients, including sugars, with excluded brands.)

**Table 1:** Nutrition statistics of brands included and excluded from analysis.

	Included Brands		Excluded Brands		
	Mean	Prop. Missing	Mean	Prop. Missing	*p*-value
Calories (kcal)	434	0.00	445	0.00	0.64
Sodium (mg)	816	0.01	936	0.03	0.09
Protein (g)	17	0.02	19	0.06	0.13
Dietary Fiber (g)	3	0.02	3	0.09	0.05
Saturated Fat (g)	7	0.01	8	0.10	0.66
Cholesterol (mg)	63	0.03	70	0.16	0.18
Sugars (g)	20	0.04	13	0.21	0.00
Calcium (dv)	23	0.22	16	0.97	0.22
Iron (dv)	11	0.22	10	0.98	0.67
Vitamin A (dv)	96	0.22	19	0.98	0.23
Vitamin C (dv)	16	0.22	33	0.98	0.31
Brands	302		427		
Stores	192,538		78,095		
Avg. stores / brand	638		183		
Med. stores / brand	62		39		

Nutrition statistics of brands included and excluded from analysis. The top panel shows the mean value of eleven nutrient attributes and the proportion of items with missing values for that attribute, with units indicated in the row label (g for grams, kcal for kilocalories, mg for milligrams, dv for proportion of daily value). The *p*-value is from a *t*-test for a mean difference between included and excluded brands, clustering at the brand level. “Brands” indicates the total number of brands (e.g., McDonald’s); “Stores” indicates the total number of physical store locations in the United States, regardless of franchise or subsidiary status; and “Avg. stores / brand” indicates the average number of store locations per brand; “Med. stores / brand” indicates the median number of store locations per brand. Store information was collected from Dun and Bradstreet and manual web searches of each individual brand. Nutrient statistics do not include items with internal macronutrient inconsistencies, brands that have no location in the United States, and brands that are misclassified as brands (e.g., packaged food with no retail locations). For details on data cleaning, see S1 Appendix Section A1 in S1 File.

To select items for analysis, we exclude beverages because they typically have minimal nutrient content [63] and children’s items because we focus only on items across which calorie disclosure will cause substitution for the modal adult consumer and we find it unlikely that adult consumers will substitute children’s menu items for adult menu items. We also exclude items listed by ingredient only, and items for bulk consumption. (Exclusions of items are not represented in Table 1 in order to maintain comparability across included and excluded brands.) We employ numerous manual and semi-automated techniques to rigorously assess and correct for inaccuracies in the data, such as rounding errors and inconsistencies within items across related nutrient inputs (e.g. saturated fat exceeding total fat). For further details on our data cleaning and validation process, see S1 File, Section A. After item exclusions, 257 brands remain for analysis. These brands represent a large swath of the chain restaurant industry: they have over 168,579 locations and include 53 of the top 100 largest brands in the U.S. in terms of sales. Because calorie disclosure may cause consumers to substitute across menu categories (pizza vs. sandwich) or within menu category (type of pizza), we classify all food items by broad menu category (appetizer, side, salad, soup, sandwich, pizza, entrée, and dessert) and subcategory. We classify desserts, for instance, into twelve subcategories (e.g., ice cream, yogurt, donut, cookie, cake, pie, brownie, etc.). For details of data classification, see Section A in S1 File. After this preprocessing, our analysis sample comprises a total of 257 brands, with all items classified.

### B. Nutrient profiling

Nutrient profile scores aggregate nutritional information of a given food item into a single score, with the aim of capturing healthfulness / nutritiousness of the item. Because there is no single universally accepted score, we consider twelve nutrient profiling systems (see S2 Fig for details on each system in S1 File). While there are differences between the inputs, the indices substantially agree on the core nutritional components and are highly correlated [64]. Moreover, because the prevalence of individual nutrient components is highly correlated across foods, model performance does not vary substantially across profiles with varying numbers of inputs [65].

**Fig 1:**
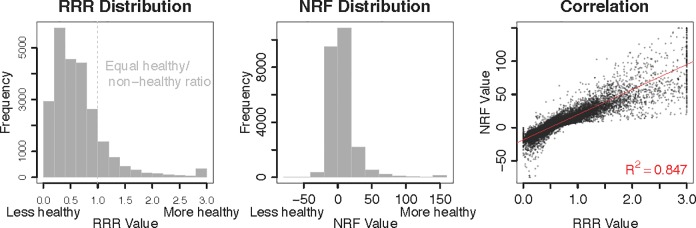
Distribution of RRR and NRF values and correlation between these indices. For visibility, RRR values are trimmed at 3 and NRF values are trimmed at -75 and 150. The red line indicates the least squares fit between RRR and NRF values (trimmed).

We focus on two profiling methods that were calculable with the available data. Scheidt and Daniel’s “Ratio of Recommended to Restricted Nutrients” (RRR), is a ratio of six recommended nutrients (relative to reference values in dietary guidelines) to five restricted nutrients (relative to reference values in dietary guidelines) per serving [19]. Scores close to zero represent lower nutrient density; scores equal to one indicate an equal proportion of recommended to restricted nutrients; and scores above one include more recommended nutrients than restricted nutrients. The left panel of **Fig 1** plots the distribution of RRR scores in brand items. For reference, we also provide RRR scores for common food items and a sample of brand items in S4 Table in S1 File, and S3 Table in S1 File depicts average RRR values by menu category and subcategory in our dataset.

The RRR index has several advantages for our study. First, in contrast to other scores, it is based on servings and can hence be adapted to the amount served by a brand. Second, because inputs are calibrated to the U.S. Dietary Guidelines and daily (reference) values (e.g., daily recommended intake of fiber is 25 grams) [19], RRR formalizes the potential tension between calorie disclosure and federal nutrition guidelines. One limitation, however, is that calories are themselves an input to the RRR score, so we may mechanistically expect some degree of correlation. We hence also calculate the RRR score excluding calories, with nearly identical results presented in S5 Table in S1 File.

The second nutrient profiling method we deploy is Fulgoni, Keast and Drewnowski’s “Nutrient Rich Foods” (NRF) score. The NRF score is calculated by subtracting the sum of the percent daily values of nutrients to limit from the sum of the percent daily values of recommended nutrients per 100 kcal (the base) [66]. Positive values indicate higher nutrient density. One advantage to the NRF score is that calories comprise the base, but are not a direct nutrient component, hence providing a measure of nutrient quality that is portion-independent. The model has been proposed using a range of inputs, hence allowing us to choose the index for which nutrient inputs are available. We use the NRF6.3 index corresponding to six nutrients to encourage and three to limit. Compared to RRR, NRF6.3 omits cholesterol, but another important difference lies in weighting and functional form, allowing us to assess sensitivity to nutrient profile method.

The limitations to NRF for our purposes are twofold. First, NRF uses “added sugars,” which are both difficult to define and calculate [67] and not reported as an independent quantity in Nutritionix data. Indeed, USDA withdrew its database of added sugar because of complications with changing formulations [68]. Nutritionix instead reports total sugar, which could include both natural and added sugars. As is commonly done [65], we hence substitute sugar for added sugar in the NRF index. Because natural sugar occurs most routinely in fruit and dairy products, while most chain restaurant food items comprise processed foods, which are the primary contributors to added sugars in the diet, we expect that total sugar of items in our dataset largely reflects added sugars [68]. Second, brands do not disclose nutrients sufficient to calculate more complex NRF models, which account for as many as 18 nutrient attributes. More complex NRF scores appear to exhibit a weaker relationship to energy density in a study of non-restaurant foods [69], which may bias our results in favor of finding an association with calories.

The middle panel of **Fig 1** depicts the distribution of NRF scores in the brand data. For reference, we also provide NRF scores for a sample of generic and brand items in S4 Table in S1 File and NRF scores by brand menu category and subcategory in our data in S3 Table in S1 File. The third panel of **Fig 1** shows that RRR and NRF scores are highly correlated (R^2^ = 0.85), even if cardinal scales differ. Because results are nearly identical, we refer mainly to the RRR results in the paper for expositional simplicity, but present full NRF results in Section D in S1 File.

### C. Methods

We model the nutrient profile score (RRR or NRF) as a function of calories for all items, and assess robustness using a series of fixed effects (linear) regressions. The sets of direct fixed effects include 257 brands, 7 menu categories, 68 menu subcategories. Brand fixed effects account for overall differences in nutrient quality across brands (e.g., McDonald’s vs. Veggie Grill). Category and subcategory fixed effects account for marginal differences in nutrient quality across categories (e.g., desserts vs. entrees) or subcategories (e.g., ice cream vs. pies amongst desserts). In each model, we use cluster-robust standard errors by brand to account for intra-brand correlation and heteroskedasticity. In the most saturated models, we control for the full interactions between brand and menu category or subcategory, so that coefficients are identified only by the correlation between calories and nutrient score within menu subcategory within a brand (e.g., the correlation of calories and RRR based on pies at Marie Callender’s). To assess sensitivity to linearity assumptions, we also fit loess models. Lastly, we also fit separate models for each category (and subcategory) to investigate heterogeneity in coefficients across menu categories.

## III Results

### A. Pooled models

**Fig 2:**
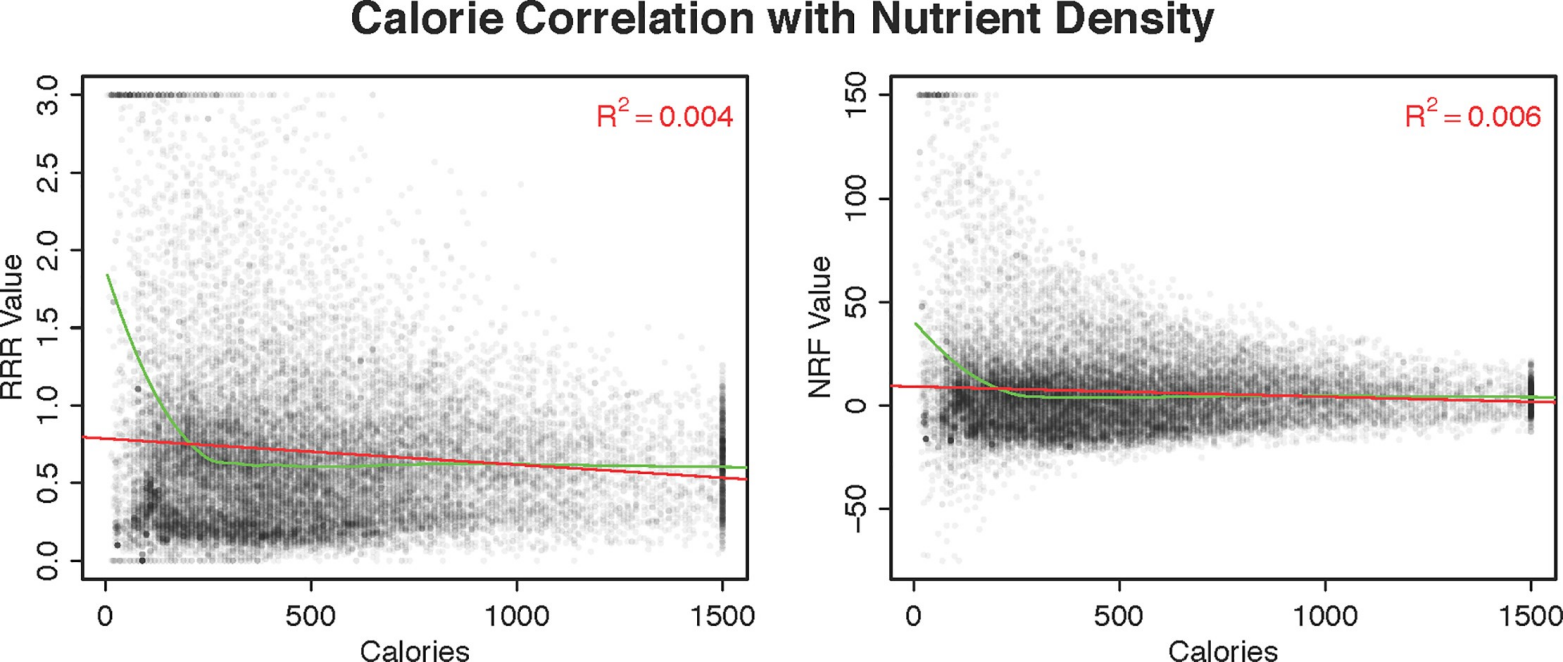
Correlation between calories on the *x*-axes and nutrient scores on the *y*-axes. Each point represents a brand food item. The left panel displays data for RRR values and the right panel displays data with NRF (per 100 kcal) values. The red line plots the raw regression line (with corresponding R^2^ value in top right corner) and the green line plots a loess fit. For visibility, data points are randomly jittered and truncated for RRR values above 3, NRF values above 150 and below -75, and calories above 1,500.

**Fig 2** presents the raw correlation between calories (on *x*-axes) and nutrient scores (on *y*-axes). Each dot represents one brand item, and the left panel plots RRR values and the right panel plots NRF values. The figure shows that there is considerable heteroskedasticity of nutrient scores in calories. This is primarily a function of sampling variability: low-calorie food items are more likely to end up with extreme measurements, as they are more likely to have exclusively recommended or exclusively restricted nutrients (e.g., a sorbet has an RRR value of 0 and a side of veggie sticks can have an RRR value exceeding 20). Put differently, while lower calorie items are significantly more likely to have high RRR values, they are also significantly more likely to have low RRR values, as can be seen by the cluster of items with calories below 400 and with RRR values of 0. The core question is whether there is, on average, a correlation in the mass of data points.

Table 2 provides regression results of RRR values. Each coefficient represents the marginal effect on the RRR score of a 100 kcal. increase. Column (A) corresponds to the red line plotted in the left panel of **Fig 2**. Results are stable across models (Columns (B)-(H)), subject to sampling variability, and we can reject the null hypothesis that there is no correlation between calories and nutrient density.

**Table 2:** Linear regression of RRR value for menu items with different fixed effects (FE) specifications.

	(A)	(B)	(C)	(D)	(E)	(F)	(G)	(H)
Calories	-0.02***	-0.03***	-0.02***	-0.02***	-0.02***	-0.01***	-0.02***	-0.02***
	(0.00)	(0.01)	(0.00)	(0.00)	(0.00)	(0.00)	(0.01)	(0.00)
Partial R^2^	0.004	0.010	0.008	0.004	0.005	0.002	0.007	0.006
Brand FE	No	Yes	No	No	Yes	Yes	Yes	Yes
Category FE	No	No	Yes	Yes	Yes	Yes	Yes	Yes
Subcategory FE	No	No	No	Yes	No	Yes	No	Yes
Brand × Categ. FE	No	No	No	No	No	No	Yes	Yes
Brand × Subcateg. FE	No	No	No	No	No	No	No	Yes
Parameters	2	258	8	64	264	320	1,043	2,256
Brands	257	257	257	257	257	257	257	257
Items	24,076	24,076	24,076	24,076	24,076	24,076	24,076	24,076

Linear regression of RRR value for menu items with different fixed effects (FE) specifications. Each top cell represents the point estimate, corresponding to the associated RRR value effect of a 100 kcal. unit increase. Standard errors, clustered at the brand level, are in parentheses below. Partial R^2^ represents the marginal variance explained by calorie count, excluding FEs. “Brand FE” control for each brand; “Categ. FE” are menu category fixed effects; “Subcategory FE” are menu subcategory fixed effects; “Brand × Categ. FE” are fixed effects for all interactions between brands and menu categories; “Brand × Subcateg. FE” are fixed effects for all interactions between brands and menu subcategories. “Parameters” indicates the total number of parameters in the linear model; “Brands” indicates the number of brands included; and “Items” represent the sample size.

*/**/*** denote statistical significance at α-levels of 0.1, 0.05, and 0.01, respectively.

The magnitude, however, is small. On average, a 100-calorie increase is associated with a 0.02-point decrease in the RRR score. For reference, substituting a glass of skimmed milk for whole milk, for instance, increases the RRR score by 0.66. The row of partial R^2^ capture the amount of variability in the RRR index that is captured by calorie counts, and it is exceedingly low: calories account for at most 1% of the variability in nutrient quality in each model.

**Fig 3:**
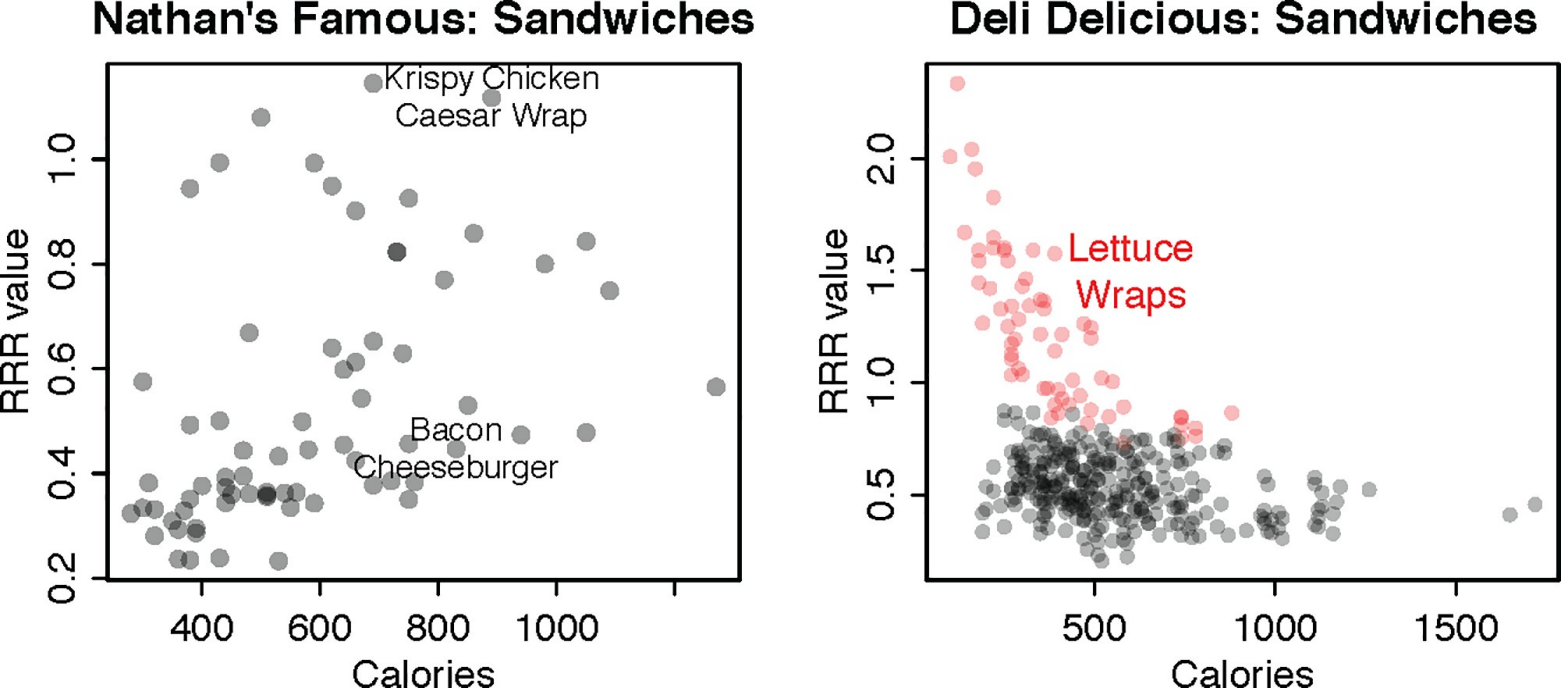
Illustration of relationship between calories and nutrient quality for sandwiches at Nathan’s Famous (left panel) and Deli Delicious (right panel).

As a concrete illustration, **Fig 3** plots the correlation for sandwiches at two brands. The left panel plots sandwiches at Nathan’s Famous, illustrating the general finding that there is no strong correlation between calories and nutrient quality. At 830 calories, the Bacon Cheeseburger has an RRR score of 0.45, but at 890 calories, the Krispy Chicken Caesar Wrap has an RRR of 1.12. If anything, at extremely low calorie counts, RRR scores appear to be worse at Nathan’s, which is driven largely by hot dogs. Yet even within the hot dog subcategory at Nathan’s, there is no statistically significant correlation between calories and RRR scores. The right panel plots a menu category with an atypically strong correlation of sandwiches at Deli Delicious. The primary reason, however, is that each sandwich can be ordered as a lettuce wrap, which are plotted in red. Excluding lettuce wraps, the correlation reduces substantially. More importantly, while the Nutritionix data includes calorie counts for each customized version of a sandwich, the actual disclosure to consumers includes only the calorie *range*. Given that the lettuce wrap anchors the low end of the range, this has the possibility of creating a false impression of healthiness relative to how items are consumed. At minimum, the range is uninformative: for the “Ham, Bacon, Sprouts, Avocado & Swiss” sandwich, for instance, the range is 420 to 1,120 calories.

### B. Heterogeneous effects

**Fig 4:**
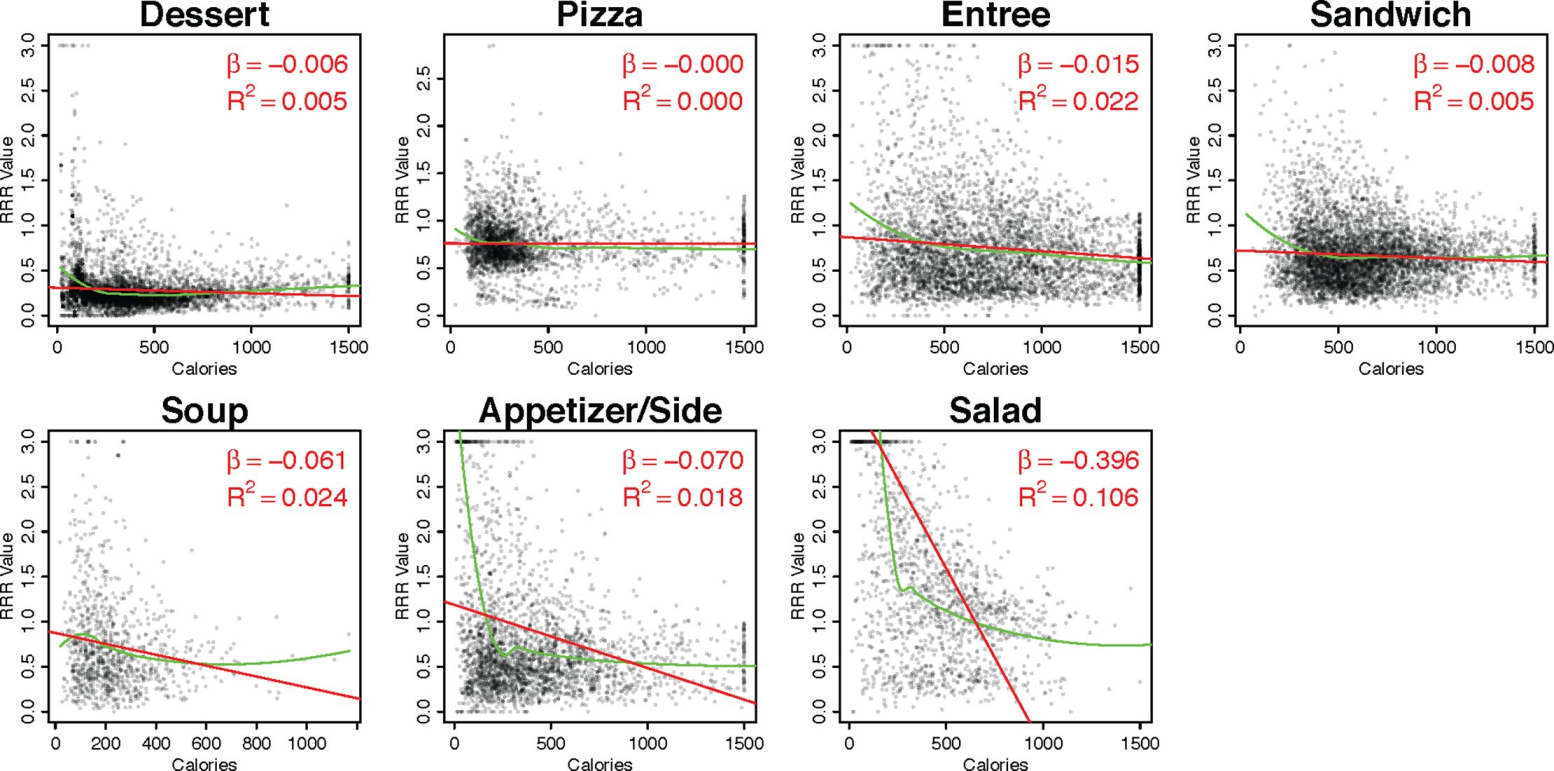
Correlation between calorie count and RRR value by main menu categories. Red lines depict the least squares fit and green lines depict a loess fit. *β* is the average increase in RRR associated with a 100 kcal. increase.

We now investigate heterogeneous effects by menu category. **Fig 4** displays the raw correlation by menu category, with panels sorted by absolute magnitude of the coefficient in a bivariate regression. For three categories, namely appetizer/sides, soups, and particularly salads, the correlation appears substantively strong.

Table 3 presents models that control for brand fixed effects, again sorted by magnitude of coefficient estimates. For most categories, the effect of calories on RRR is limited, with some even displaying a positive relationship between the two. For salads, a 100-calorie increase is associated with an appreciable 0.35 decrease, plus or minus 0.1 at the 95% level, in RRR scores. The complexity with the salad interpretation, however, is that (a) starter and side salads represent the cluster of items with low calorie counts and high RRR scores, and (b) brands vary in whether dressings are included in calorie counts. For example, in forty-three instances, brands listed Caesar salads without dressings, creating the impression of a lower calorie count, even though Caesar salads are unlikely to be ordered with any other dressing. We hence coded whether salads were disclosed with or without dressings and run separate models for this of 458 main salads. We find that the correlation is substantially (and statistically significantly) weaker for dressed main salads: a 100-calorie increase is associated with a 0.07-point decrease in the RRR score. In other words, while salads appear to be the best exemplar for calories signaling nutrient quality, the correlation in Table 3 may not reflect how items are actually consumed.

**Table 3:** Regression of marginal effect of 100 kcal increase on RRR score separate for each main menu category.

	Dessert	Pizza	Entree	Sandwich	Appetizer/Side	Soup	Salad
Calories	0.01**	0.00	-0.01***	-0.02***	-0.08***	-0.11***	-0.35***
	(0.00)	(0.00)	(0.00)	(0.00)	(0.02)	(0.02)	(0.07)
Partial R^2^	0.003	0.006	0.019	0.037	0.018	0.077	0.072
Brand FE	Yes	Yes	Yes	Yes	Yes	Yes	Yes
Brands	186	70	180	168	193	102	146
Items	5,774	2,869	4,605	5,602	2,805	1,007	1,414

Regression of marginal effect of 100 kcal increase on RRR score separate for each main menu category. Each cell represents point estimate with standard errors, clustered by brand, in parentheses, controlling for brand fixed effects. Partial R^2^ represents the variance explained by calorie count.

*/**/*** denote statistical significance at α-levels of 0.1, 0.05, and 0.01, respectively. *P*-values are adjusted using the Benjamini Hochberg procedure [70].

### C. Robustness

We report a range of robustness tests in the Section C in S1 File. First, recall that calories are one of the restricted nutrient inputs to RRR. We hence re-run all of the models re-calculating the RRR index to exclude calories (Section C.1 in S1 File). Second, we rule out that portion size–which would affect caloric content, but not nutrient density–accounts for our findings, by subsetting to items with a fixed portion size (Section C.2 in S1 File). Third, because nutrient scores can take on extreme values in low ranges of calories, we re-fit models after trimming the values of outliers (Section C.3 in S1 File). Fourth, due to potential inaccuracies in missing value codes applied to fiber, iron, vitamin A, and vitamin C, we re-run regressions excluding items we predict to be affected (Section C.4 in S1 File). Fifth, RRR and NRF call for nutrient values to be capped at 100% daily value, causing some items to have overvalued indices, we thus re-run regressions without capping nutrient values (Section C.5 in S1 File). Sixth, because it is possible that the correlation is stronger with more fine-grained menu categories, we estimate separate models for each menu subcategory (e.g., for dessert muffins) (Section C.6 in S1 File). Seventh, we conducted the full set of analyses with NRF scores (Section D in S1 File). Results remain comparable across all of these robustness checks. Last, we assess whether our data may be confounded by direct effects of the calorie labeling requirement on the formulation of menu items because our data postdates the announcement of the requirement in the 2010 ACA. Prior studies have documented reductions in calories with MenuStat data [60,71]. To assess this possibility, we sample menu items and compare calorie counts before and after the enactment of the labeling requirement. We do not find strong evidence that brands reduced the calories of their menu items, likely because the data pre-date the effectiveness of the labeling requirement.

## IV. Limitations

We note several limitations with our data analysis. First, our sample does not include all chain brands covered by the ACA, so our findings may not generalize to all covered brands. Moreover, even included brands may provide requisite nutritional information for only a subset of items. For instance, there are 26 brands for which fewer than five items are disclosed with information requisite to calculate RRR. That said, we do not find evidence that included and excluded brands differ substantially on observable nutrient components (Table 1), so it is unlikely that items at omitted brands would exhibit a stronger correlation between calories and nutrient quality. Moreover, the in-sample effect remains relevant for a substantial part of the restaurant sector.

Second, Nutritionix is based on self-reporting by brands, so we have no independent verification of the validity of the nutrient component data. One study of nearly 270 food items at 42 restaurants found that while stated energy contents were accurate overall, there were substantial inaccuracies for individual foods, with nearly 20% understating energy by more than 100 kcal [72]. We similarly discovered a number of potential inaccuracies. For instance, sugars exceeded the number of total carbohydrates for some items. For some brands, fiber, vitamin A, vitamin C, and iron are all listed as zero, but ingredients suggest that these components should have been coded as missing values. We document these in Section C.4 in S1 File and show that our results do not appear affected by these detectable inaccuracies. Nonetheless, measurement error that we have not detected may attenuate the correlation. However, because the information brands report to Nutritionix is the same information consumers would receive upon request at a brand location, to the extent there are inaccuracies, such inaccuracies reflect the broader limitations of a brittle disclosure regime that limits consumers in obtaining accurate information.

Third, there may also be measurement error in menu categorization of items. While we developed semi-automated coding schemes based on item name, descriptions, and nutrient components, in consultation with copies of menus available online to classify menu items (see Section A.2 in S1 File), it can remain difficult to classify all items correctly. For instance, a “low carb wrap” might refer to a full food item or only an ingredient. To address this concern, we conducted a full manual check of classifications of all items in all brands to reduce any measurement error, comparing items against online menus. We then blindly classified a sample of 300 items, finding accuracy to exceed 95%.

Last, while our study informs the tension within food law and the scope of disclosure, our inquiry cannot speak deeply to the broader criticism that diet quality should focus on foods, not specific nutrient inputs [23]. Our study does support the view, however, that fixating on singular attributes (or “nutritionism” as popularized by Michael Pollan [73]) can come with its limits.

## V. Discussion

We now spell out five legal and policy implications of our work.

First, our study clarifies the rationale for calorie disclosure. Our findings reject the notion that calories can meaningfully signal nutrient quality. The remaining coherent justifications for calorie disclosures strike us as threefold. As a matter of nutritional science, calories may simply be the most important attribute, although, as noted above, this is a matter of some dispute [13,20]. Our findings show that there is no easy resolution to this dispute for disclosure of calories at chain restaurants. A second rationale is that while substituting toward lower-calorie items may not improve nutrient quality, calorie disclosure may deter individuals from eating at restaurants [26]. To date, we are not aware of rigorous evidence of this behavioral mechanism. A third rationale is that calorie disclosure–even if it alone will achieve few public health benefits–is a first strategic step for more transparency in the food system, given that restaurants were categorically exempt under the Nutrition Labeling and Education Act prior to the ACA. Perhaps observing calories will increase consumer demand for more comprehensive nutrition information.

Second, because calories do not signal overall healthfulness, our findings suggest that greater attention should be paid to micronutrient disclosure in addition to calories. ACA requirements to make available a limited set of micronutrients upon request is a positive step in this direction, but *none* of the twelve nutrient profiles could be calculated if nutrient information were limited to that required under the FDA’s menu labeling regulation. The FDA could strengthen the information basis considerably by requiring that all micronutrients for conventional nutrient profiles be disclosed, including at minimum calcium, iron, vitamin A, and vitamin C. Indeed, FDA already possesses the legal authority to do so [2]. Our research indicates that such an approach would impose limited costs on consumers and on restaurants. With respect to consumers, expanding disclosure of requisite nutrient attributes is not inherently inconsistent with simplification if it enables calculation of summary nutrient profile measures. For example, France adopted a simple color-coded “Nutri-score” system based on the fuller nutrient profile; and Massachusetts General Hospital engaged in color-coding of the overall healthfulness, not just calories, of food items in its cafeteria with evident success [74,75]. With respect to restaurants, while costs were hotly debated with FDA’s proposed menu labeling rule [76], our research also suggests that the *marginal* cost of disclosing additional nutrient components would be very low. The reason is that laboratories commonly base calorie counts on recipes submitted by restaurants. And recipe analysis simply draws on ingredients (e.g., tomatoes in the USDA Food Composition Database) with standard nutrient output beyond calories.

Third, our research suggests that FDA should require such information to be disclosed under a uniform microdata standard, as contemplated under Executive Order 13642 [77]. Replacing the currently inconsistent dissemination, a uniform microdata standard would allow consumers, nutritionists, restaurant sites, technologists, and researchers could more easily study, rate, and compare items, both within and between establishments and customize disclosures for subpopulations with different nutrient needs (e.g., increasing the weight of iron and folate for pregnant women [78], increasing the weight of calcium for children [79]). Comprehensive disclosure would also facilitate research, validation, and development of evidence-based disclosure metrics.

Fourth, our research highlights considerable discretion for restaurants in implementing calorie disclosure. For example, for items that give customers multiple topping or flavor options (“variable menu items”), regulations require restaurants list calories separately for toppings and “basic preparation,” without defining the latter [80]. Restaurants appear to use such discretion to create the impression of lower calorie counts (e.g., exclusion of dressing for a Caesar salads). Discretion in toppings should not be an avenue to skirt disclosure. Similarly, regulations allow restaurants to post calorie ranges instead of the calories of each option separately [80]. Deli Delicious’s calorie range of a single sandwich from 420 to 1,120, for instance, provides consumers with little usable information. More meaningful for consumers would be to require “build-as-you-go” brands to disclose ingredient calories prominently along with the range.

Finally, disclosure is weak without enforcement [4]. Regulations require establishments to have a “reasonable basis” for establishing the calorie and nutritional content of their offerings [80]. The most common method for establishing nutrient values is by sending recipes to laboratories, but calculating nutrients based on ingredient lists may not accurately reflect the way dishes are usually prepared or ordered. The data integrity issues we discovered (e.g., an item with 1,216 grams of fiber and a total of 80 grams of carbohydrates) suggest that validation is necessary. Absent more serious enforcement, such as random sampling by FDA and validation protocols by laboratories, consumers should be aware that calorie and nutrition disclosures are subject to considerable uncertainty.

In sum, our paper illustrates the possibility for so-called Goodhart’s law in action: when a measure becomes a target, it may undermine the measure. Just as 20th century low-fat fixation may have distorted dietary quality [81], if calories are not the single most important nutrient attribute, calorie disclosure may distort nutrient quality. Of course, the same concern would apply to disclosure based on nutrient profile indices. Consumption based exclusively on nutrient profile scores (e.g., consuming only spinach) may ignore balance and variety of dietary intake. And fortification solely to improve the nutrient profile could invalidate the measure as a target. Ultimately, our research highlights the limits of one disclosure metric alone.

## Supporting information

S1 File (DOCX)
