# Supplementary material for: Menu labeling, calories, and nutrient density: Evidence from chain restaurants
Source: PLoS One. 2020 May 7;15(5):e0232656. doi: 10.1371/journal.pone.0232656 (PMC7205205; doi:10.1371/journal.pone.0232656)
Supplement: S1 File — (DOCX) [file pone.0232656.s001.docx]

**Supporting Information**

**Menu labeling, calories, and nutrient density: Evidence from chain restaurants**

Daniel E. Ho^[[1]](#footnote-1)*^, Oluchi Mbonu^[[2]](#footnote-2)^*,* Anne McDonough,^[[3]](#footnote-3)^ Rebecca Pottash^[[4]](#footnote-4)^

Contents

A. Data Cleaning and Item Classification 2

1. Cleaning 2

2. Classification 3

B. Nutrient profiles 8

1. Twelve nutrient profiling systems 8

2. Reference values for RRR and NRF profiling methods 9

C. Robustness Checks with RRR Scores 10

1. Excluding Calories from Index 10

2. Excluding Varying Portion Sizes 11

3. Trimming Caloric Outliers 12

4. Imputations of Iron, Vitamin A, Vitamin C, and Calcium 13

5. Uncapping Nutrient Values 14

6. Subcategory Regressions 15

7. Brand size 17

D. NRF Regression Results 19

E. Establishments Included 21

## A. Data Cleaning and Item Classification

### Cleaning

After we subset the data down to establishments that contain at least one item with requisite nutrition information to calculate our nutrient profile scores, we further cleaned the data as follows. First, we manually identify and exclude forty-three establishments for one of four reasons: (i) nutrition information is only provided for ingredients (e.g., Chipotle lists caloric counts of black beans); (ii) the brand is misclassified as a restaurant (e.g., Go Bites is a nutrition bar company); (iii) the brand is no longer in operation; or (iv) the brand has no locations in the United States. Second, we remove ingredients across all brands, using manual and semi-automated approaches (e.g., regular expressions for “add-on” in the item description). Papa John’s, for instance, includes “BBQ Sauce” as a menu item. Third, we remove items that are intended for consumption by more than one individual using manual and semi-automated approaches (e.g., regular expression for “to share,” “bulk item,” “for two”). For instance, Tubby’s has a garden salad described as “party size.” Fourth, we remove drinks, food items which include drinks, and children’s items, again using manual and semi-automated techniques. Fifth, we identify a series of internal inconsistencies for macronutrients. Protein inconsistencies occur, for instance, when total calories from protein exceed total calories by more than some threshold. Thresholds for macronutrient inconsistencies are built to account for possible rounding errors. Rounding rules for declaring nutrients are obtained from FDA regulations [1]. Last, we identified several unit inconsistencies. For instance, 54th Street Grill & Bar’s “California Bleu Chicken Salad,” is listed has having a daily value percentage of 10,000, but the restaurant website clarifies that this is in International Units, which we converted to daily values. This unit inconsistency occurred primarily for iron, calcium, vitamin A and vitamin C values. Table 1 summarizes the scope of this data cleaning by the number of brands and the number and percentage of items affected.

|  | Brands | Items | Affected % | Resolution |
| --- | --- | --- | --- | --- |
| Drink | 176 | 13,108 | 25.3 | Removed |
| Excluded Brands | 43 | 6,976 | 13.5 | Removed |
| Ingredients | 221 | 6,340 | 12.3 | Removed |
| Children’s food | 121 | 1,406 | 2.7 | Removed |
| Bulk Items | 40 | 797 | 1.5 | Removed |
| Carbohydrate inconsistencies | 49 | 112 | 0.2 | Removed |
| Fat inconsistencies | 40 | 129 | 0.2 | Corrected |
| Unit inconsistencies | 5 | 127 | 0.2 | Removed |
| Protein inconsistencies | 3 | 5 | 0.0 | Removed |

Table 1: Summary of data cleaning. “Drinks” are beverages and menu items which contain beverages; “Excluded Brands” are establishments that were removed because only ingredients were listed, the brand is misclassified as a restaurant, the brand is no longer in operation, or the brand has no U.S. locations; “Ingredients” are only components of menu items as conventionally understood; “Children’s food” are items on a children’s menu; “Bulk Items” are meant for consumption by two or more people; “Fat Inconsistencies” occur when (a) calories from fat exceed total calories by more than 20kcal, (b) saturated fat exceeds total fat by more than 2g, (c) trans fats exceed total fat by more than 2g, and/or (d) total fat calories exceeds total calories by more than 20kcal; “Unit inconsistencies” occur when nutrients are not in the unit specified in the data dictionary and have to be converted; “Carbohydrate Inconsistencies” occur when (a) sugar exceeds total carbohydrates by more than 2g, (b) dietary fiber exceeds total carbohydrates by more than 2g, and/or (c) calories from carbohydrates exceed total calories by more than 20kcal; “Protein Inconsistencies” occur when calories from protein exceed total calorie count by more than 20 kcal. Thresholds for fat, carbohydrate and protein inconsistencies account for possible rounding errors, based on FDA regulations [2]. Groups are not mutually exclusive and were removed/corrected in the following order: Excluded Brands, Ingredients, Bulk Items, Drinks, Carbohydrate Inconsistencies, Protein Inconsistencies, Fat Inconsistencies, Children’s food, and Unit Inconsistencies.

### Classification

To classify items into menu categories and subcategories, we develop a series of regular expressions for item names and descriptions. We search for key words in both the item name and item description and use matched terms to categorize the item (e.g. an item with “sandwich” in its name and/or description is likely a sandwich). In the event of a conflict, i.e. an item matches to more than one category, we resolve the categorization manually. For example - “Peanut Butter Cookie Sandwich” matches as both a Sandwich and a Dessert, but is categorized as Dessert. Table 2 summarizes the search terms used to categorize, and sub-categorize items. All items not categorized through regular expressions were categorized manually.

| **Category** | **Sub-Category** | **Key Words** |
| --- | --- | --- |
| Appetizer/Side |  | Appetizer, Antipasto, Antipasti, Opener, Starter, Sashimi, Potsticker, Dolma, Deviled Eggs, Crab Rangoon, Crab Dip, Gyoza, Samosa, Side, Chips, Rings Tots, Bites, Cheesesticks, Breadsticks, Potato salad, Macaroni salad, Pasta salad, Poutine, Okra, [!french]Toast, [!with, !without, !no, !banana, !pumpkin]Bread |
|  | Wings | Wings[!salad, !sauce] |
|  | Calamari | Calamari |
|  | Nachos | Nachos |
|  | Garlic Bread | Garlic Toast, Garlic Cheese Toast, Three-Cheese Toast, Garlic Bread, Garlic Knots, Garlic Baguette |
|  | Fries | [!with, !without, !w/, !w/o]Fries, Fryz |
|  | Chips | [!with, !without, !w/, !w/o, !fish and]Chips |
|  | Dip | Dip |
|  | Hummus | Hummus |
|  | Egg/Spring Rolls | Egg Roll, Spring Roll |
|  | Bread/Cheese Sticks | Stick |
|  | Rice | Rice |
|  | Fruit Salad | [!with, !without, !add, !no, !punch]Fruit Salad[!punch, !blast] |
|  | Vegetables | Broccoli, Veggies, Green Beans, Cauliflower, Kale, Sprouts, Vegetables, Asparagus, Slaw, Carrot |
|  | Beans | Beans |
|  | Potato | Potato, Hush Puppies |
| Salad |  | [!pasta, !potato, !macaroni, !tortellini, !rice]Salad[!dressing, !sandwich], Insalata, Ensalada, Cobb, Insalate, Lettuce Wedge |
|  | Side Salad | Salad + Side |
|  | Starter Salad | Salad + Starter, Salad +Appetizer |
|  | Undressed | Add {desc} dressing, no dressing, without dressing, dressing not included |
|  | Dressed | With {desc} dressing, With {desc} Vinaigrette, With {desc} Sauce, With ranch |
| Soup |  | Soup, Bisque, Chowder, Soupe, Cream of…, Borscht |
|  | Side Soup | Soup + Side |
|  | Starter Soup | Soup + Starter, Soup +Appetizer |
| Entrée |  | Entrée, Main Course, Stir Fry, Bowl, Lunch, Combo, Dinner, Meal, Pot roast, Plate, Fajita, Falafel, Lamb, Turkey, Casserole, Sopapilla, Carnitas, Fried Rice, Loin, Potato, Chop, Carne, Platter, Parmigiana, Quiche, Curry, Sushi, Piri, Tempura, Bulgogi, Meat, Loaf, Crab, Tostada |
|  | Pasta | Pasta, Lasagna, Spaghetti, Ziti, Penne, Rigatoni, Ravioli, Mac, Fettuccini, Dan Dan, Lo mein, Pad thai, Yakisoba, Fusilli, Linguini, Rotini, Pinwheels, Gnocchi, Macaroni, Alfredo |
|  | Breakfast | Oatmeal[!bar], Pancakes, Waffle[!fries], Scrambler, Scrambled, Omelet(te), Frittata, Benedict, French Toast, Breakfast, [!tea]Biscuit |
|  | Burrito | Burrito, Quesarito, Bar-rito |
|  | Taco | Taco |
|  | Quesadilla | Quesadilla |
|  | Calzone | Calzone |
|  | Enchilada | Enchilada |
|  | Steak | Sirloin, Filet Mignon, New York strip, New York striploin, Rib eye, Prime rib, Rib steak, T-bone, Tri-tip, Flat iron |
|  | Ribs | Ribs |
|  | Tofu | Tofu |
|  | Shrimp | Shrimp |
|  | Fish | Fish, Tilapia, Salmon, Mahi, Cod, Tuna, Trout |
|  | Chicken | Chicken[!noodle] |
| Sandwich |  | Grilled cheese, Melt, Pastrami, Croissant, Cheesesteak, Big, Sloppy, French Dip, Monte Cristo |
|  | Wrap | Wrap |
|  | Submarine | Submarine, Grinder[!dough], Hoagie, Sub, Hero, footlong |
|  | Burger | Burger[!sauce, !dill, !stroganoff, !soup], Slider, Hambunger (spelling error on purpose), Double-Double |
|  | Hot Dog | Hot Dog |
|  | Bagel | Bagel, Squagel |
|  | Breakfast | Biscuit, Muffin, Breakfast, Brekwich, Egg (if already categorized as sandwich) |
|  | Panini | Panini, Gyro, Pita[!bread] |
|  | Traditional | [!for small, !for medium, !for large]Sandwich[!extra], Melt, Sandwich, Club, Reuben |
| Pizza |  | Pizza, Flatbread |
|  | Slice | (Search in serving unit and serving qty)  Serving unit – Slice  Serving qty – 1 |
| Dessert/Pastries |  | Sorbet, Flan, Malt[!vinegar], Cannoli, Pizookie Baklava, Churros, Cinnapie, S’more, Danish, Macaroon, Éclair, Crème Brulee, Cream Puffs, Pudding, Munchkin, Treat, Banana Split, Creamz, Cinnamon Roll, Apple Fritter, Scone, Dessert |
|  | Yogurt | Yoghurt, Yogurt, Joegurt |
|  | Cake | [!Crab]Cake, Tiramisu |
|  | Ice Cream | Soft Serve, Ice Cream, Sundae, Sherbet, Ice Dream. Sorbet, Frozen Custard, Gelato, Sorbeto, Italian Ice, Gelati, Cone |
|  | Cookie | Cookie |
|  | Float | Float |
|  | Pie | [!pot]Pie[!pancake], Cobbler, Tart(e), Torte |
|  | Donut | Donut, Doughnut, Cruller |
|  | Pretzel | Pretzel |
|  | Muffin | [!english]Muffin |
|  | Brownie | Brownie |
|  | Mousse | Mousse |
|  | Dessert Pizza | Dessert Pizza, Pizzert |

Table 2: Key words used in regular expressions for categorization and subcategorization of data items. In all instances, key words include singular and plural. The “!” sign indicates that regular expressions was fine-tuned to exclude specific combination of terms (e.g. “cake” preceded by “crab” is not a dessert; “salad” preceded by “pasta” is not a salad). Based on manually checking all brands, we also allowed for brand-specific terms (e.g., “Fryz” at Sheetz are “Fries,” “Bar-ritos” at Protein Bar are “Burritos”). Any item containing multiple items from different categories (e.g., “sandwich with fries”) is categorized as an entrée. Salad subcategories are not mutually exclusive (e.g., salads can be both dressed and sides).

We leveraged several other sources of information for classification. First, Nutritionix contains an optional field for serving size units. For example, some sandwiches have a serving size unit of “Sandwich,” some pizzas have a unit of “slice,” and some salads have a unit of “Salad.” This field was particularly helpful in cases were neither the item name nor description adequately characterized the item. For instance, an item named “Chicken Salad” and serving unit “Sandwich” allowed us to recognize this item as a chicken salad sandwich. In addition, the field permitted us to identify items that included sides/salads. For example, an item with name “pastrami burger,” but with serving unit “burger with sides” was classified as an entrée category. Second, for 36 restaurants, Nutritionix provides establishment-specific menu categorizations. While these menu categorizations do not map perfectly to ours, they were useful in providing consistent classifications. For example, Firehouse Subs has several menu items with names like “Turkey – Medium” and “Chicken Breast – Small,” but the Firehouse menu category “Single Meat Subs” indicates that these are sandwiches.

Even with these automated classifications, our process required substantial manual resolution. For instance, key terms are often not unique to a menu category. The term “chili,” for instance, can create false positives for soup (e.g., “Fritos Chili Pie, Small,” “Chili Cheese Tots, Mini,” “Chili Cheese Loaded Potatoes”), so we manually categorized these items. Similarly, the term “biscuit” is categorized by default as a sandwich (e.g., “Chicken biscuit,” “Sausage, Egg & Cheese Biscuit”), but plain biscuits (e.g., biscuits with jam) were manually re-categorized as sides. Other examples of such terms are “Croissants” (falling either in the sandwich or dessert (pastry) category) and “Potatoes” (sides or part of entrée).

One particularly challenging category was “Wings.” Shane’s Rib Shack, for instance, has a 6-piece wing item, while Pizza Inn offers a 20-piece wing option. We used a cutoff of eight wings to distinguish appetizers from entrée, which is consistent with conventional understandings of portion sizes [2–4][3–5]. Some establishments explicitly label the number of wings in the item name, description, or serving size unit, making this task easy. For others, it is more difficult to infer. We hence fit a regression of number of wings against calories in the sample where both are observed and predict wing counts for brands where only calories are observed. Fig 1 displays the data, finding an eight-wing cutoff of roughly 722kcal. (For robustness, we also use cutoffs of 4, 5, 6, and 12 wings, with no significant change in results.

Fig 1: Plot of total wing count versus total caloric count for those wing dishes with an explicit wing count in the item name, description, or serving size unit. The red line depicts a least squared fit of wing count against calorie count.

Last, one of the major difficulties in classifications was in distinguishing ingredients. Because ingredients are described in a wide range of ways (e.g., “BBQ sauce,” “Ham for Mountain Pizza,” “Meatball Sandwich without bread, big,” “Wrap”), we conducted a comprehensive manual brand-by-brand check. We examined names, unusually low-calorie items, items with incongruous serving units (e.g., “1 tbsp”), inconsistent calorie distributions (especially within categories), and inconsistent naming conventions within categories and establishments, and online menus to determine when an ingredient was listed. Blind categorization of a random sample of 300 items revealed an accuracy rate greater than 95% in menu categorization.

Table 3 reports the subcategories, number of brands, items, and calories within each category.

**Table 3**: Summary statistics of food items by menu category

| **Category** | **Subcategories** | **Brands** | **Items** | **Calories** | **RRR Value** | **NRF Value** |
| --- | --- | --- | --- | --- | --- | --- |
| **Appetizer/ Side** | Wings, Fries, Potato, Veggie, Nachos, Rice, Garlic Bread, Beans, Calamari, Dip, Fruit Salad, Hummus, Chips, Egg Rolls, Cheese/Bread Stick, Garlic Toast, Other | 193 | 2,805 | 431 | 0.89 | 10 |
| **Salad** | Starter / side Salad (dressed / undressed), Other (dressed/ undressed) | 146 | 1,414 | 451 | 1.79 | 44 |
| **Soup** | Side / Starter Soup, Other | 102 | 1,007 | 233 | 0.73 | 5 |
| **Entree** | Chicken, Breakfast, Pasta, Wings, Burrito, Taco, Fish, Shrimp, Steak, Ribs, Calzone, Quesadilla, Enchilada, Tofu, Other | 180 | 4,605 | 800 | 0.75 | 9 |
| **Sandwich** | Sub, Traditional, Burger, Breakfast, Wrap, Bagel, Hot Dog, Panini, Other | 168 | 5,602 | 640 | 0.67 | 6 |
| **Pizza** | Slice, Other | 70 | 2,869 | 471 | 0.76 | 8 |
| **Dessert** | Ice Cream, Yogurt, Donut, Cookie, Cake, Pie, Muffin, Brownie, Dessert Pizza, Pretzel, Float, Mousse, Other | 186 | 5,774 | 394 | 0.28 | -7 |

Table 3: Summary statistics of food items by menu category. “Brands” indicates the number of brands with an item under the classification row and “Items” indicate the total number of items. “Calories,” “RRR value” and “NRF Value” indicate the mean calorie count, RRR index, and NRF index for each item category. RRR refers to the “Ratio of Recommended to Restricted Nutrients” profiling method, and NRF refers to the “Nutrient Rich Foods” profiling method. To convey prevalence, subcategories excluding “Other” are sorted by decreasing sample size.

## B. Nutrient profiles

### Twelve nutrient profiling systems

We consider twelve nutrient profiling systems depicted in Fig 2. Each row indicates one nutrient profiling system with colored cells indicating which nutrient inputs are required, with nutrients split by whether consumption is recommended on the left (in green) or to be restricted on the right (in red) and ordered by commonality from left to right within these groups. The grey bars behind nutrient labels indicate the proportion of items in the Nutritionix data that disclose the nutrient. While there are considerable differences between the inputs, the indices are highly correlated. Moreover, because the prevalence of individual nutrient components is highly correlated across foods, model performance does not vary substantially across profiles with varying numbers of inputs [60]. Scarborough et al. surveyed 700 nutrition professionals to rank healthiness of 120 food items, and found that all of the seven indices had correlation coefficients with these rankings between 0.62 to 0.80 [61]. Fig 2 also illustrates that there is substantial agreement across these indices around core nutritional components: seven indices rely on the first five recommended components, which are also the components most frequently disclosed in the Nutritionix data.

**Figure 2:** Nutrient inputs for twelve nutrient profiling methods
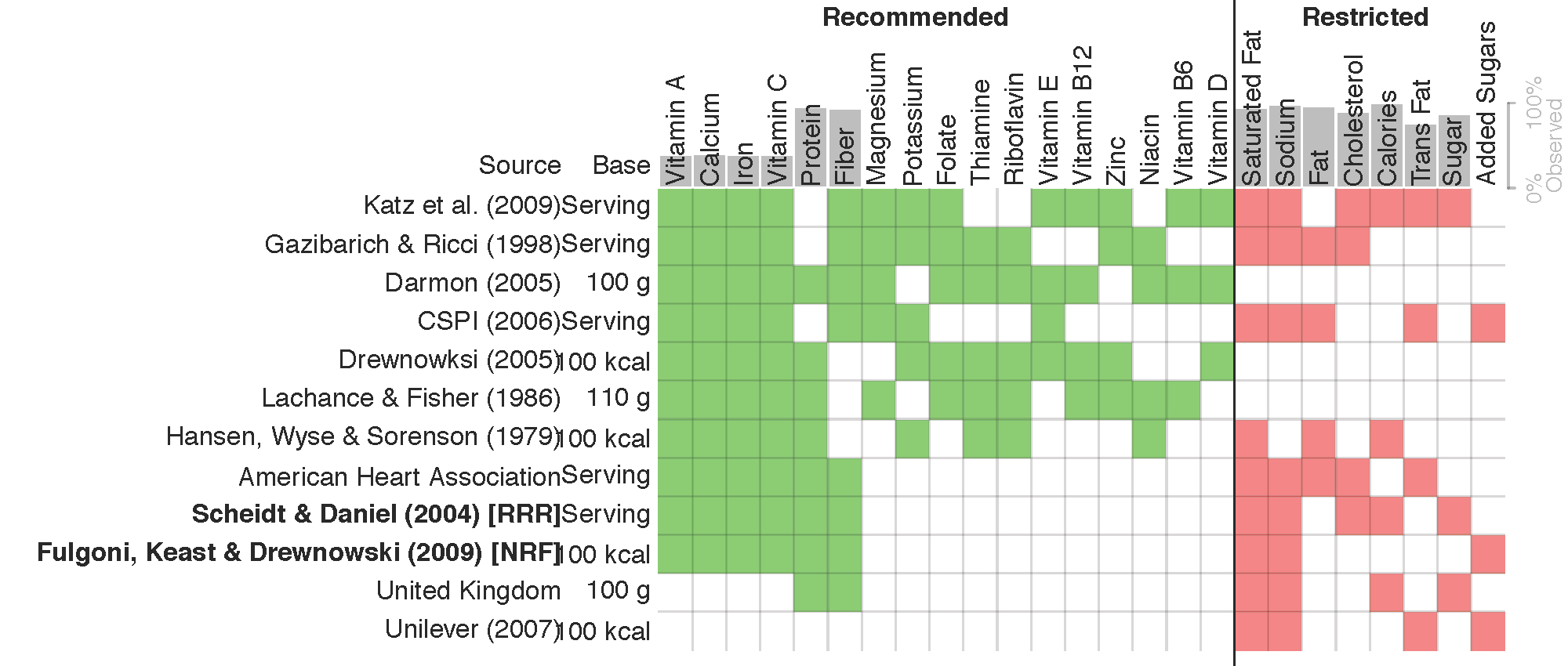


**Fig 2: Nutrient inputs for twelve nutrient profiling methods**. Rows represent nutrient profile scores (ordered in decreasing order of the number of inputs) and columns represent nutrient attributes. Cells are colored when the nutrient attribute is an input for the specific index. The left portion depicts nutrients for which increasing consumption is generally recommended (in green) and the right portion depicts nutrients for which restricting consumption is advised (in red), and within these categories nutrient attributes are ordered by how commonly the input is used. Nutrients are depicted if included in more than two indices. Grey bars behind nutrient attribute labels indicate the percentage of items containing that nutrient attribute in the Nutritionix data (excluding closed, non-USA and non-restaurant establishments). Fulgoni, Keast & Drewnowski depict the NRF6.3 index. In contrast to other nutrient scoring systems, the American Heart Association and CSPI (Center for Science in the Public Interest) use a binary indicator based on nutrient thresholds, rather than calculating a continuous score. Hansen, Wyse & Sorenson calculate multiple scores for each food item corresponding to distinct nutrients.

### Reference values for RRR and NRF profiling methods

For reference, Table 4 provides RRR and NRF scores for common food items and a sample of restaurant items. One medium carrot has an RRR index of nearly 13; a serving of chips has an RRR index of 0.86; and a glazed donut has an RRR score of 0.1. Substituting a glass of skimmed for whole milk results in an improvement of 0.66 on the RRR scale. On the NRF scale, raw carrots, for instance, have an NRF score of 451, while chips have an NRF score of 6.

**Table 4**: RRR and NRF scores for select generic and branded food items

|  |  | **Item** | **Cal.** | **RRR** | **NRF** |
| --- | --- | --- | --- | --- | --- |
| *Generic food* |  | Raw carrots (1 medium) | 25 | 12.97 | 451 |
|  |  | Raw broccoli (1 serving) | 50 | 10.94 | 301 |
|  |  | Orange | 45 | 4.30 | 217 |
|  |  | Milk, skimmed (8 oz) | 83 | 1.26 | 50 |
|  |  | Milk, whole (8 oz) | 149 | 0.60 | 10 |
|  |  | Chips (1 oz) | 149 | 0.86 | 6 |
| *Branded food* | Gordon Biersch | Salad (house, tangy vinaigrette) | 270 | 2.44 | 58.13 |
|  | CA Pizza Kitchen | Pizza (five cheese and tomato) | 270 | 0.71 | 6.59 |
|  | Winchell’s | Donut (french glazed) | 270 | 0.10 | -15.5 |

Table 4: RRR and NRF scores for select generic and branded food items. “Cal.” indicates calories. Nutrient information for generic food items comes from the USDA Food Composition Database Release 28, using items 11124 (Carrots, raw), 11090 (Broccoli, raw), 09200 (Oranges, raw, all commercial varieties), 01085 (Milk, nonfat, fluid, with added vitamin A and vitamin D), 01077 (Milk, whole, 3.25% milkfat, with added vitamin D), and 19411 (Snacks, potato chips, plain, salted).

## C. Robustness Checks with RRR Scores

Here we present a series of robustness checks with RRR scores as the outcome.

### Excluding Calories from Index

As noted above, the fact that calories are included in the RRR index might induce a correlation when other nutrient components might be orthogonal to calories. We hence re-calculate the RRR index by excluding calories from the denominator. Table 5 presents regression estimates comparable to those in Table 2 of the main paper. If anything, the correlation grows slightly in magnitude, but the results are substantially the same. A 100-calorie increase is associated with a 0.02 decrease in RRR scores and the proportion of variability explained by calories is at 0.003 or below for all specifications.

|  | (A) | (B) | (C) | (D) | (E) | (F) | (G) | (H) |
| --- | --- | --- | --- | --- | --- | --- | --- | --- |
| Calories | -0.02^***^ | -0.04^***^ | -0.03^***^ | -0.02^***^ | -0.03^***^ | -0.02^***^ | -0.03^***^ | -0.02^***^ |
|  | (0.01) | (0.01) | (0.01) | (0.01) | (0.01) | (0.00) | (0.01) | (0.01) |
|  |  |  |  |  |  |  |  |  |
| Partial R^2^ | 0.002 | 0.003 | 0.002 | 0.001 | 0.001 | 0.001 | 0.002 | 0.001 |
| Brand FE | No | Yes | No | No | Yes | Yes | Yes | Yes |
| Category FE | No | No | Yes | Yes | Yes | Yes | Yes | Yes |
| Subcategory FE | No | No | No | Yes | No | Yes | No | Yes |
| Brand × Categ. FE | No | No | No | No | No | No | Yes | Yes |
| Brand × Subcateg. FE | No | No | No | No | No | No | No | Yes |
| Parameters | 2 | 258 | 8 | 64 | 264 | 320 | 1,043 | 2,250 |
| Brands | 257 | 257 | 257 | 257 | 257 | 257 | 257 | 257 |
| Items | 24,051 | 24,051 | 24,051 | 24,051 | 24,051 | 24,051 | 24,051 | 24,051 |

Table 5: Linear regression of RRR value, excluding calories as an input, for menu items with different fixed effects (FE) specifications. All other elements are the same as in Table 2 of the main paper. Sample sizes are slightly lower because RRR indices are undefined when calories are excluded.

### Excluding Varying Portion Sizes

Nutrition indices are designed to remain near constant with varying portion sizes – RRR

is a ratio of nutrient densities, and NRF scales nutrients to a base of 100kcal. In contrast, calories vary directly with portion size. An increase in portion size for example, will increase calorie count but not the nutrition index. The concern is that the weak correlation between the nutrition indices and caloric content may be driven by varying portion sizes of the same menu item. We hence investigate the sensitivity of our results to different portion sizes. First, we randomly sample 50 restaurants. This sample includes 8,115 menu items. Second, we manually identify different portion sizes of the same menu item and focus only on one portion size (the smallest). For example, Carvel (an ice cream franchise) has a small vanilla ice cream cup, and a large vanilla ice cream cup. We retain only the small cup size. Note that not all menu items have varying portion sizes, and we do not consider substitutions or slight variations to be a different portion size. For instance, we include the small ‘Carvelite’ vanilla ice cream cup (‘Carvelite is a trademarked low-fat alternative) as we consider it to be a unique menu item (and not a varying portion size). We are left with 5,797 menu items. Third, we re-estimate the regressions with our new dataset of unique portion sizes. Table 6 presents results with no substantial differences in portion size.

|  | (A) | (B) | (C) | (D) | (E) | (F) | (G) | (H) |
| --- | --- | --- | --- | --- | --- | --- | --- | --- |
| Calories | -0.02^**^ | -0.05^***^ | -0.04^***^ | -0.03^***^ | -0.04^***^ | -0.03^***^ | -0.05^***^ | -0.03^***^ |
|  | (0.01) | (0.01) | (0.01) | (0.01) | (0. 01) | (0. 01) | (0. 01) | (0. 01) |
|  |  |  |  |  |  |  |  |  |
| Partial R^2^ | 0.004 | 0.014 | 0.011 | 0.005 | 0.008 | 0.003 | 0.011 | 0.012 |
| Brand FE | No | Yes | No | No | Yes | Yes | Yes | Yes |
| Category FE | No | No | Yes | Yes | Yes | Yes | Yes | Yes |
| Subcategory FE | No | No | No | Yes | No | Yes | No | Yes |
| Brand × Categ. FE | No | No | No | No | No | No | Yes | Yes |
| Brand × Subcateg. FE | No | No | No | No | No | No | No | Yes |
| Parameters | 2 | 51 | 8 | 61 | 57 | 110 | 234 | 536 |
| Brands | 50 | 50 | 50 | 50 | 50 | 50 | 50 | 50 |
| Items | 5,797 | 5,797 | 5,797 | 5,797 | 5,797 | 5,797 | 5,797 | 5,797 |

Table 6: Linear regression of RRR value, removing items that are simply a larger portion size of another existing item, with different fixed effects (FE) specifications. All other elements are the same as in Table 2 of the main paper.

### Trimming Caloric Outliers

Some restaurants offer items that are very high in calories and, as explained above, the RRR index can exhibit high variability at low caloric counts. We hence investigate the sensitivity of our results to such outliers by truncating calories at 1500 and RRR scores at 3. Table 7 presents results, with substantially comparable results.

|  | (A) | (B) | (C) | (D) | (E) | (F) | (G) | (H) |
| --- | --- | --- | --- | --- | --- | --- | --- | --- |
| Calories | -0.01^***^ | -0.02^***^ | -0.02^***^ | -0.02^***^ | -0.02^***^ | -0.02^***^ | -0.02^***^ | -0.02^***^ |
|  | (0.00) | (0.00) | (0.00) | (0.00) | (0.00) | (0.00) | (0.00) | (0.00) |
|  |  |  |  |  |  |  |  |  |
| Partial R^2^ | 0.005 | 0.026 | 0.024 | 0.015 | 0.020 | 0.013 | 0.027 | 0.021 |
| Brand FE | No | Yes | No | No | Yes | Yes | Yes | Yes |
| Category FE | No | No | Yes | Yes | Yes | Yes | Yes | Yes |
| Subcategory FE | No | No | No | Yes | No | Yes | No | Yes |
| Brand × Categ. FE | No | No | No | No | No | No | Yes | Yes |
| Brand × Subcateg. FE | No | No | No | No | No | No | No | Yes |
| Parameters | 2 | 258 | 8 | 64 | 264 | 320 | 1,043 | 2,256 |
| Brands | 257 | 257 | 257 | 257 | 257 | 257 | 257 | 257 |
| Items | 24,076 | 24,076 | 24,076 | 24,076 | 24,076 | 24,076 | 24,076 | 24,076 |

Table 7: Linear regression of RRR value, truncating caloric values at 1,500 and RRR values at 3, for menu items with different fixed effects (FE) specifications. All other elements are the same as in Table 2 of the main paper.

As an additional check, we also run the re-estimate the regression by removing all items with a calorie count greater than 1500, and/or an RRR value greater than 3. Note that this estimation removes very healthy menu items with large RRR values, e.g. a side of broccoli. Table 8 shows these results.

|  | (A) | (B) | (C) | (D) | (E) | (F) | (G) | (H) |
| --- | --- | --- | --- | --- | --- | --- | --- | --- |
| Calories | -0.00 | -0.02^***^ | -0.02^***^ | -0.01^***^ | -0.02^***^ | -0.01^***^ | -0.02^***^ | -0.02^***^ |
|  | (0.00) | (0.00) | (0.00) | (0.00) | (0.00) | (0.00) | (0.00) | (0.00) |
|  |  |  |  |  |  |  |  |  |
| Partial R^2^ | 0.000 | 0.013 | 0.015 | 0.010 | 0.014 | 0.010 | 0.019 | 0.014 |
| Brand FE | No | Yes | No | No | Yes | Yes | Yes | Yes |
| Category FE | No | No | Yes | Yes | Yes | Yes | Yes | Yes |
| Subcategory FE | No | No | No | Yes | No | Yes | No | Yes |
| Brand × Categ. FE | No | No | No | No | No | No | Yes | Yes |
| Brand × Subcateg. FE | No | No | No | No | No | No | No | Yes |
| Parameters | 2 | 257 | 8 | 64 | 263 | 319 | 1,038 | 2,184 |
| Brands | 256 | 256 | 256 | 256 | 256 | 256 | 256 | 256 |
| Items | 23,046 | 23,046 | 23,046 | 23,046 | 23,046 | 23,046 | 23,046 | 23,046 |

Table 8: Linear regression of RRR value, removing items with caloric values greater than 1,500 and/or RRR values greater than 3, for menu items with different fixed effects (FE) specifications. All other elements are the same as in Table 2 of the main paper.

### Imputations of Iron, Vitamin A, Vitamin C, and Calcium

FDA’s menu labeling rule, in contrast to the Nutrition Labeling and Education Act does not require establishments to disclose iron, vitamin A, and vitamin C, and Calcium (IACC). As can be gleaned from Table 1, even amongst the set of included establishments, these four nutrients are often missing for roughly 22% of items. In spot-checking the data, we noticed that there appears to be a cluster of establishments with high caloric counts, but zero values for these four nutrients. We hypothesized that these constitute false zero values that should actually be missing value codes. For example, Brixx Wood Fired Pizza’s “Caribbean Jerk Chicken Pizza, Wheat Dough” lists values for zero for all four nutrients, but the restaurant’s own online nutrition information lists 10% of the daily value of iron for the item. We further hypothesized that this was an establishment specific phenomenon. Fig 3 plots the total number of items on an establishment’s menu against the proportion of its items that have values of zero for all four nutrients. The distribution is bimodal, with a cluster of establishments for which nearly all items have values of zero for these four nutrients (false zeroes). We hence re-estimate the regression by omitting such items if an establishment had more than 10% of items with zero IACC. Table 9 presents results, showing no substantial differences.

Fig 3: Seeming confusion between zero-values and missing data codes for iron, vitamin A, vitamin C, and Calcium.

|  | (A) | (B) | (C) | (D) | (E) | (F) | (G) | (H) |
| --- | --- | --- | --- | --- | --- | --- | --- | --- |
| Calories | -0.02^***^ | -0.03^***^ | -0.02^***^ | -0.02^***^ | -0.02^***^ | -0.02^***^ | -0.03^***^ | -0.02^***^ |
|  | (0.00) | (0.01) | (0.01) | (0.00) | (0.01) | (0.00) | (0.01) | (0.01) |
|  |  |  |  |  |  |  |  |  |
| Partial R^2^ | 0.003 | 0.011 | 0.008 | 0.004 | 0.006 | 0.003 | 0.008 | 0.007 |
| Brand FE | No | Yes | No | No | Yes | Yes | Yes | Yes |
| Category FE | No | No | Yes | Yes | Yes | Yes | Yes | Yes |
| Subcategory FE | No | No | No | Yes | No | Yes | No | Yes |
| Brand × Categ. FE | No | No | No | No | No | No | Yes | Yes |
| Brand × Subcateg. FE | No | No | No | No | No | No | No | Yes |
| Parameters | 2 | 240 | 8 | 64 | 246 | 302 | 931 | 1,948 |
| Brands | 239 | 239 | 239 | 239 | 239 | 239 | 239 | 239 |
| Items | 21,025 | 21,025 | 21,025 | 21,025 | 21,025 | 21,025 | 21,025 | 21,025 |

Table 9: Linear regression of RRR value for menu items with different fixed effects (FE) specifications, excluding items (from establishments with greater than 10% of total items with zero IACC) where iron, vitamin A, vitamin C and Calcium jointly equal zero. All other elements are the same as in Table 2 of the main paper.

### Uncapping Nutrient Values

Both RRR and NRF call for nutrients to be capped at 100% daily value. This ensures that items with a large amount of only one nutrient, or items that have been artificially fortified are not overvalued [6,7]. However, this truncation of nutrients causes some high calorie items to be overvalued. In particular, desserts with higher calories tend to contain more than 100% of the daily recommended value of sugar. Because their sugar content is thus capped at 100%, these desserts appear to have less sugar per calorie than their smaller portion (and lower calorie) equivalencies - sometimes registering them as healthier. This nutrient cap is the main driving factor behind the positive correlation between calories and RRR for the dessert category (without capping nutrients, the estimate becomes -0.00). We hence re-estimate our regression without capping nutrient values at 100% dv. Table 10 presents these results.

|  | (A) | (B) | (C) | (D) | (E) | (F) | (G) | (H) |
| --- | --- | --- | --- | --- | --- | --- | --- | --- |
| Calories | -0.03^***^ | -0.05^***^ | -0.03^***^ | -0.03^***^ | -0.04^***^ | -0.03^***^ | -0.04^***^ | -0.03^***^ |
|  | (0.01) | (0.01) | (0.01) | (0.01) | (0.01) | (0.01) | (0.01) | (0.01) |
|  |  |  |  |  |  |  |  |  |
| Partial R^2^ | 0.004 | 0.008 | 0.005 | 0.003 | 0.005 | 0.003 | 0.006 | 0.004 |
| Brand FE | No | Yes | No | No | Yes | Yes | Yes | Yes |
| Category FE | No | No | Yes | Yes | Yes | Yes | Yes | Yes |
| Subcategory FE | No | No | No | Yes | No | Yes | No | Yes |
| Brand × Categ. FE | No | No | No | No | No | No | Yes | Yes |
| Brand × Subcateg. FE | No | No | No | No | No | No | No | Yes |
| Parameters | 2 | 258 | 8 | 64 | 264 | 320 | 1,043 | 2,256 |
| Brands | 257 | 257 | 257 | 257 | 257 | 257 | 257 | 257 |
| Items | 24,076 | 24,076 | 24,076 | 24,076 | 24,076 | 24,076 | 24,076 | 24,076 |

Table 10: Linear regression of RRR value for menu items with different fixed effects (FE) specifications. Nutrient values are not capped at 100% of the daily value for any item. All other elements are the same as in Table 2 of the main paper.

### Subcategory Regressions

For expositional simplicity, we present only category regressions above. Here, we present regression results for the top 9 subcategories (within categories) with sample sizes exceeding 100 items. While highly sophisticated consumers might draw conditional inferences about the informational signal of calories based on these subcategory results, the more direct way to transmit this information would be to convey nutrient quality directly. Across all of these subcategories except for salads, effect sizes remain substantively small. In desserts, higher calories are associated with higher RRR scores across six subcategories.

|  | **Entree** |  |  |  |  |  |  |  |  |
| --- | --- | --- | --- | --- | --- | --- | --- | --- | --- |
|  | Shrimp | Wings | Taco | Other | Burrito | Pasta | Chicken | Fish | Breakfast |
| Calories | -0.00 | -0.01^***^ | -0.01 | -0.02 | -0.02 | -0.02^**^ | -0.02^**^ | -0.03^***^ | -0.05^**^ |
|  | (0.00) | (0.00) | (0.01) | (0.01) | (0.01) | (0.01) | (0.01) | (0.01) | (0.02) |
|  |  |  |  |  |  |  |  |  |  |
| Partial R^2^ | 0.007 | 0.080 | 0.009 | 0.013 | 0.010 | 0.039 | 0.030 | 0.129 | 0.052 |
| Brand FE | Yes | Yes | Yes | Yes | Yes | Yes | Yes | Yes | Yes |
| Brands | 42 | 33 | 35 | 124 | 22 | 74 | 119 | 50 | 64 |
| Items | 189 | 424 | 240 | 1,091 | 301 | 485 | 652 | 211 | 574 |
|  |  |  |  |  |  |  |  |  |  |
|  | **Sandwich** |  |  |  |  |  |  |  |  |
|  | Hot Dog | Sub | Breakfast | Other | Traditional | Burger | Panini | Wrap | Bagel |
| Calories | 0.01 | -0.01^**^ | -0.02 | -0.02^***^ | -0.02^***^ | -0.03^***^ | -0.04 | -0.06 | -0.10^***^ |
|  | (0.01) | (0.01) | (0.01) | (0.01) | (0.01) | (0.01) | (0.02) | (0.03) | (0.03) |
|  |  |  |  |  |  |  |  |  |  |
| Partial R^2^ | 0.023 | 0.041 | 0.038 | 0.032 | 0.033 | 0.091 | 0.036 | 0.101 | 0.191 |
| Brand FE | Yes | Yes | Yes | Yes | Yes | Yes | Yes | Yes | Yes |
| Brands | 26 | 38 | 58 | 69 | 119 | 85 | 25 | 69 | 27 |
| Items | 124 | 1,511 | 526 | 546 | 1,414 | 730 | 107 | 439 | 205 |
|  |  |  |  |  |  |  |  |  |  |
|  | **Dessert** |  |  |  |  |  |  |  |  |
|  | Yogurt | Ice Cream | Brownie | Other | Donut | Cake | Pie | Muffin | Cookie |
| Calories | 0.02 | 0.01^***^ | 0.01 | 0.00 | 0.00 | 0.00^***^ | -0.00 | -0.01 | -0.02 |
|  | (0.01) | (0.00) | (0.01) | (0.00) | (0.00) | (0.00) | (0.00) | (0.03) | (0.02) |
|  |  |  |  |  |  |  |  |  |  |
| Partial R^2^ | 0.002 | 0.043 | 0.067 | 0.003 | 0.003 | 0.059 | 0.000 | 0.006 | 0.052 |
| Brand FE | Yes | Yes | Yes | Yes | Yes | Yes | Yes | Yes | Yes |
| Brands | 55 | 60 | 57 | 114 | 19 | 56 | 44 | 28 | 92 |
| Items | 1,221 | 1,862 | 102 | 859 | 517 | 266 | 229 | 158 | 353 |
|  |  |  |  |  |  |  |  |  |  |
|  | **Appetizer/Side** | |  |  |  | **Salad** |  | **Pizza** |  |
|  | Fries | Other | Wings | Potato | Veggie | Other | App/Side Salad | Other | Slice |
| Calories | -0.02 | -0.04^**^ | -0.06 | -0.09 | -1.48^**^ | -0.27^***^ | -1.63 | 0.00 | -0.01 |
|  | (0.01) | (0.01) | (0.06) | (0.06) | (0.62) | (0.05) | (1.06) | (0.00) | (0.01) |
|  |  |  |  |  |  |  |  |  |  |
| Partial R^2^ | 0.028 | 0.011 | 0.052 | 0.018 | 0.170 | 0.072 | 0.171 | 0.009 | 0.001 |
| Brand FE | Yes | Yes | Yes | Yes | Yes | Yes | Yes | Yes | Yes |
| Brands | 91 | 173 | 51 | 64 | 68 | 144 | 75 | 51 | 34 |
| Items | 355 | 1,402 | 364 | 172 | 156 | 1,255 | 159 | 866 | 2,003 |
|  |  |  |  |  |  |  |  |  |  |

Table 11: Regression estimates for each menu subcategory. Each cell represents coefficient for 100 kcal increase with robust standard errors clustered by establishment in parentheses. All models control for brand fixed effects. NRF is measured per 100kcal. ^*^/^**^/^***^ denote statistical significance at α-levels of 0.1, 0.05, and 0.01, respectively. P-values are adjusted using the Benjamini Hochberg procedure [8].

### Brand size

While menu items are identical for the same chain across locations, item availability and consumption may differ geographically. Although data limitations preclude an analysis of brand-geographic variation, here we explore variation on a related brand characteristic that is available in our data: brand size. Smaller brands will typically be more geographically concentrated, and an observable implication of the geographic hypothesis might be that we should see a stronger correlation in smaller brands. We stratify our sample on the number of brand locations, grouping our data into the following quantiles: “small” (items at brands with fewer than 22 locations); “mid-size” (22-60 locations); “large” (61-299 locations); “largest” (300+). We then re-run our main analyses on each subset. The results, which are presented below in Table 12, indeed suggest that calories and nutrient density exhibit a relatively stronger correlation in small brands compared to larger brands. That said, even in small chains, the relationship remains weak.

|  | (A) | (B) | (C) | (D) | (E) | (F) | (G) | (H) |
| --- | --- | --- | --- | --- | --- | --- | --- | --- |
| **Small**  Calories | -0.04^***^ | -0.08^***^ | -0.07^***^ | -0.06^***^ | -0.07^***^ | -0.05^***^ | -0.07^***^ | -0.06^***^ |
| Partial R^2^  Brands  Items | (0.02)  0.009  64  4,645 | (0.02)  0.025  64  4,645 | (0.01)  0.024  64  4,645 | (0.01)  0.017  64  4,645 | (0.02)  0.017  64  4,645 | (0.01)  0.009  64  4,645 | (0.02)  0.019  64  4,645 | (0.01)  0.023  64  4,645 |
|  |  |  |  |  |  |  |  |  |
| **Mid-size**  Calories  Partial R^2^  Brands  Items  **Large**  Calories  Partial R^2^  Brands  Items  **Largest**  Calories    Partial R^2^  Items  Brands | -0.02^***^  (0.01)  0.010  64  6,848  -0.01  (0.01)  0.001  64  6,136  -0.01  (0.01)  0.005  65  6,447 | -0.03^***^  (0.01)  0.015  64  6,848  -0.02^**^  (0.01)  0.004  64  6,136  -0.01  (0.01)  0.004  65  6,447 | -0.02^***^  (0.01)  0.010  64  6,848  -0.02^**^  (0.01)  0.004  64  6,136  -0.01  (0.01)  0.004  65  6,447 | -0.02^***^  (0.00)  0.006  64  6,848  -0.01  (0.01)  0.001  64  6,136  -0.01  (0.01)  0.002  65  6,447 | -0.02^***^  (0.00)  0.007  64  6,848  -0.02^**^  (0.01)  0.003  64  6,136  -0.01  (0.01)  0.002  65  6,447 | -0.01^***^  (0.00)  0.003  64  6,848  -0.01  (0.01)  0.001  64  6,136  -0.01  (0.00)  0.001  65  6,447 | -0.03^***^  (0.01)  0.013  64  6,848  -0.02^**^  (0.01)  0.004  64  6,136  -0.01  (0.01)  0.001  65  6,447 | -0.02^***^  (0.01)  0.008  64  6,848  -0.01^**^  (0.01)  0.003  64  6,136  -0.01  (0.01  0.002  65  6,447 |
| Brand FE | No | Yes | No | No | Yes | Yes | Yes | Yes |
| Category FE | No | No | Yes | Yes | Yes | Yes | Yes | Yes |
| Subcategory FE | No | No | No | Yes | No | Yes | No | Yes |
| Brand × Categ. FE | No | No | No | No | No | No | Yes | Yes |
| Brand × Subcateg. FE | No | No | No | No | No | No | No | Yes |

Table 12: Linear regression of RRR value for menu items with different fixed effects (FE) specifications by brand size. We stratify our sample by brand size quantile, which results in the following subclasses: “Small”: brands with fewer than 22 locations; “Mid-size”: 22-60 locations; “Large”: 61-299; “Largest”: 300+. Each top cell represents the point estimate, corresponding to the associated RRR value effect of a 100 kcal. unit increase. Standard errors, clustered at the brand level, are in parentheses below. Partial R^2^ represents the marginal variance explained by calorie count, excluding FEs. “Brand FE” control for each brand; “Categ. FE” are menu category fixed effects; “Subcategory FE” are menu subcategory fixed effects; “Brand × Categ. FE” are fixed effects for all interactions between brands and menu categories; “Brand × Subcateg. FE” are fixed effects for all interactions between brands and menu subcategories. “Parameters” indicates the total number of parameters in the linear model; “Brands” indicates the number of brands included; and “Items” represent the sample size. ^*^/^**^/^***^ denote statistical significance at α-levels of 0.1, 0.05, and 0.01, respectively.

## D. NRF Regression Results

We now present the same regression estimates using NRF6.3 as the outcome variable. For interpretation, it is important to keep in mind that the absolute scale for NRF scores differs, which can be seen in Fig 1 of the main paper. Results, presented in Table 13, are comparable. A 100-calorie increase is associated with between a 0.5 to 0.8 decrease in the NRF score, when the difference between whole and skimmed milk is roughly 40 points on the NRF scale. As with RRR results, partial R^2^ remains below 0.02 across all specifications.

|  | (A) | (B) | (C) | (D) | (E) | (F) | (G) | (H) |
| --- | --- | --- | --- | --- | --- | --- | --- | --- |
| Calories | -0.49^***^ | -0.87^***^ | -0.78^***^ | -0.60^***^ | -0.74^***^ | -0.53^***^ | -0.77^***^ | -0.63^***^ |
|  | (0.12) | (0.14) | (0.14) | (0.11) | (0.13) | (0.09) | (0.15) | (0.11) |
|  |  |  |  |  |  |  |  |  |
| Partial R^2^ | 0.006 | 0.016 | 0.015 | 0.008 | 0.012 | 0.006 | 0.013 | 0.011 |
| Brand FE | No | Yes | No | No | Yes | Yes | Yes | Yes |
| Category FE | No | No | Yes | Yes | Yes | Yes | Yes | Yes |
| Subcategory FE | No | No | No | Yes | No | Yes | No | Yes |
| Brand × Categ. FE | No | No | No | No | No | No | Yes | Yes |
| Brand × Subcateg. FE | No | No | No | No | No | No | No | Yes |
| Parameters | 2 | 258 | 8 | 64 | 264 | 320 | 1,043 | 2,256 |
| Brands | 257 | 257 | 257 | 257 | 257 | 257 | 257 | 257 |
| Items | 24,076 | 24,076 | 24,076 | 24,076 | 24,076 | 24,076 | 24,076 | 24,076 |

Table 13: Linear regression of NRF value for menu items with different fixed effects (FE) specifications. Each top cell represents the point estimate, corresponding to the associated NRF value effect of a 100 kcal unit increase. Standard errors, clustered at the establishment level, are in parentheses below. Partial R^2^ represents the marginal variance explained by calorie count, excluding FEs. “Brand FE” control for each brand / establishment; “Categ. FE” are menu category fixed effects; “Subcategory FE” are menu subcategory fixed effects; “Brand × Categ. FE” are fixed effects for all interactions between establishments and menu categories; “Brand × Subcateg. FE” are fixed effects for all interactions between establishments and menu subcategories. “Parameters” indicates the total number of parameters in the linear model; “Brands” indicates the number of brands included; and “Items” represent the sample size. ^*^/^**^/^***^ denote statistical significance at α-levels of 0.1, 0.05, and 0.01, respectively.

Table 14 presents NRF regression results by category. Again, substantive effects remain small, with the largest effects being in side, soup, and salad menu categories.

|  | Dessert | Pizza | Entree | Sandwich | Appetizer/ Side | Soup | Salad |
| --- | --- | --- | --- | --- | --- | --- | --- |
| Calories | 0.24 | -0.02 | -0.57^***^ | -0.77^***^ | -2.08^***^ | -4.41^***^ | -9.83^***^ |
|  | (0.15) | (0.06) | (0.10) | (0.21) | (0.38) | (1.20) | (1.63) |
|  |  |  |  |  |  |  |  |
| Partial R^2^ | 0.004 | 0.000 | 0.044 | 0.040 | 0.028 | 0.047 | 0.120 |
| Brand FE | Yes | Yes | Yes | Yes | Yes | Yes | Yes |
| Brands | 186 | 70 | 180 | 168 | 193 | 102 | 146 |
| Items | 5,774 | 2,869 | 4,605 | 5,602 | 2,805 | 1,007 | 1,414 |

Table 14: Regression of marginal effect of 100 kcal increase on NRF score separate for each main menu category. Each cell represents point estimate with standard errors, clustered by establishment, in parentheses, controlling for brand fixed effects. Partial R^2^ represents the variance explained by calorie count. ^*^/^**^/^***^ denote statistical significance at α-levels of 0.1, 0.05, and 0.01, respectively. P-values are adjusted using the Benjamini Hochberg procedure [8].

## E. Establishments Included

| 7-11 | Chick-fil-A | Grotto Pizza | NRgize | Start Restaurant |
| --- | --- | --- | --- | --- |
| 16 Handles | Chickpea | Hale and Hearty Soups | Oberweis Dairy | Steak N' Shake |
| 54th Street Grill & Bar | Chili's | Happy Joe's | Old Chicago | Subway |
| A&W Restaurants | Chipotle | Hoss's Family Steak And Sea House | Olive Garden | Surf City Squeeze |
| Amato's Sandwich Shop | Chop't | Humdingers | On The Border Mexican Grill & Cantina | Swirlberry |
| Ameci Pizza & Pasta | Chronic Tacos | IHOP | Ono Hawaiian Bbq | T.MAC |
| America's Taco Shop | Cinnabon | In-N-Out Burger | Pancheros | Taco Bell |
| American Deli | Claim Jumper | Ivar's | Panera Bread | Taco Del Mar |
| American Flatbread | Coffee Beanery | Jack Astor's Bar & Grill | Papa John's | TacoTime |
| Anderson's Frozen Custard | Coffee Culture | Jamba Juice | Papa Murphy's | Tasti D-Lite |
| Apollo Burger | Cold Stone Creamery | Jason's Deli | Papa's Pizza To Go | Teriyaki Madness |
| Arby's | Cook Out | Jersey Mike's Subs | Pasha's | Texas Roadhouse |
| Aroma Joe's | Cosi | Jet's Pizza | Pepperjax Grill | TGI Friday's |
| Asian Chao | Costco Food Court | Jimmy John's | Perkins Restaurant And Bakery | The Cheesecake Factory |
| Atlanta Bread | Country Kitchen | Jimmy's Egg | Piada Italian Street Food | The Greene Turtle Sports Bar & Grille |
| Au Bon Pain | Cracker Barrel | Joe's Crab Shack | Pinkberry | The Habit Burger Grill |
| Auntie Anne's | Dairy Queen | Joey Restaurant Group | Pizza Inn | The Hungry Hobo |
| Austin's Pizza | Deli Delicious | Joey's Seafood | Pizza My Heart | The Melt |
| Back Yard Burgers | Denny's | Jreck Subs | Pizza Nova | The Nutrition Factory |
| Bahama Buck's | Desert Moon Grille | Juice It Up | Pizza Shoppe | The Old Spaghetti Factory |
| Baja Fresh | DiBella's Subs | Just Fresh | Pizza Studio | The Pickle Barrel |
| Bar-B-Cutie | Dickey's Barbecue Pit | Just Salad | PizzaRev | Thundercloud Subs |
| Baskin Robbins | Dilly's Sandwiches | Kahala Coffee Traders | Pj's Coffee & Tea | Tijuana Flats |
| Bd's Mongolian Grill | District Taco | Kokoro Restaurant | Planet Smoothie | Tim Horton's |
| Bellacino's Pizza & Grinders | Donatos | Krispy Kreme | Planet Wings | Tom and Chee |
| Bigfoot Java | Duchess Shoppe | Kwik Trip | Portillo's | Tony Roma's |
| Bill Miller Bar-B-Q | Duck Donuts | La Madeleine | Potbelly | Toojay's Gourmet Deli |
| Biscuitville | Dunkin' Donuts | Ledo Pizza | Pretzelmaker | Torchy's Tacos |
| Blimpie | Dunn Brothers Coffee | Lion's Choice | Protein House | Tropical Smoothie Cafe |
| Blizz Frozen Yogurt | East Side Mario's | Little Caesars Pizza | Purebread Deli | Tubby's |
| Bob Evans | Einstein Bros. Bagels | Lone Star Steakhouse & Saloon | Qdoba | Tully's Coffee |
| Boloco | El Torito | Long John Silver's | Quaker Steak & Lube | Uno Chicago Grill |
| Boneheads | Elephant Bar | Luby's | Red Lobster | Urbane Cafe |
| Bono's Pit Bar-B-Q | Erbert & Gerbert's | Mad Mex | Red Mango | Veggie Grill |
| Booster Juice | Famous Dave's | MaggieMoo's Ice Cream & Treatery | Red Robin | Waba Grill |
| Boston Market | Fields Good Chicken | Magic Wok | Redbrick Pizza | Ward's Restaurants |
| Boston Pizza | Figaro's Italian Pizza | Mama Fu's | Rice King | Wawa |
| Braum's | Firebirds Wood Fired Grill | Marble Slab Creamery | Rio Grande Mexican Restaurant | Wayback Burgers |
| Breadsmith | Firehouse Subs | Marco's Pizza | Rita's Ice | Wendy's |
| Brixx Wood Fired Pizza | First Watch | Margaritaville | Roti | Western Sizzlin |
| Broadway Pizza Restaurant | Food Exchange | Marie Callender's | Roy Rogers Restaurants | Wg Grinders |
| Brown Bag | Foster's Grille | Maui Wowi Hawaiian Coffee & Smoothies | Russo's New York Pizzeria | Whataburger |
| Bruster's Real Ice Cream | Freddy's Frozen Custard & Steakburgers | McDonald's | Rusty Bucket Restaurant And Tavern | White Castle |
| Buca Di Beppo | Freshens | Mcmenamins | Salad Creations | Wienerschnitzel |
| Buddy's Bar-b-q | Freshii | Meatheads Burgers & Fries | Salata | Winchell's Donut House |
| Buffalo Wild Wings | Friendly's | Merzi | Samuel Mancino's Italian Eatery | Wing Zone |
| Buffalo Wings & Rings | Frullati Cafe & Bakery | Milio's Sandwiches | Samurai Sam's Teriyaki Grill | Winger's Grill & Bar |
| Burger King | Furr's Family Dining | Mission BBQ | Sansai Japanese Grill | Wings To Go |
| California Pizza Kitchen | Gatti's Pizza | Moe's Southwest Grill | Second Cup | Wok Box |
| California Tortilla | Glory Days Grill | Mountain Mike's Pizza | Shane's Rib Shack | World of Beer |
| Caribou Coffee | Golden Krust Caribbean Bakery & Grill | Moxie Java | Shari's Restaurant & Pie Bakery | Wrapido |
| Carvel | Golden Spoon Frozen Yogurt | Moxie's Classic Grill | Sheetz | Yogli Mogli |
| Casey's General Stores | Good Times Burgers & Frozen Custard | Mrs. Fields | Silver Mine Subs | Yogurt Mountain |
| Cereality | Goodcents | My Friend's Place | Simple Simon's Pizza | Yogurtland |
| Champagne French Bakery Caf√© | Gordon Biersch Brewery | Nalley Fresh | Sizzler | Yoshinoya |
| Charley's Grilled Subs | Graeter's Ice Cream | Nando's Peri-Peri | Smoothie King | Yum-yum Donuts |
| Charlie Brown's Steakhouse | Great American Cookies | Nathan's Famous | Snappy Tomato Pizza | Zaxby's |
| Cheddar's Scratch Kitchen | Great Harvest Bread Co. | Nestle Tollhouse Cafe | Sonic | Zoup! |
| Cheeburger Cheeburger | Great Steak | Nick-N-Willy's Pizza | Sonny's Real Pit Bar-b-q |  |
| Cheezie's Pizza | Great Wraps | Ninety Nine Restaurants | Souplantation & Sweet Tomatoes |  |
| Chester's | Green Beans Coffee | Noble Roman's Pizza | Starbucks |  |

**REFERENCES**

1. Nutrition Labeling of Food.

2. Food and Drug Administration. Food Labeling; Nutrition Labeling of Standard Menu Items in Restaurants and Similar Retail Food Establishments. Fed Regist. 2014 Dec 1;79(230):71156–71259.

3. Kraft Heinz. Appetizer math [Internet]. Recipes. [cited 2018 Feb 7]. Available from: www.kraftrecipes.com/recipes/holidays-and-entertaining/planning/appetizer-math.aspx

4. Chihak S. Party appetizers and calculator [Internet]. Better Homes and Gardens. 2015 [cited 2018 Feb 7]. Available from: www.bhg.com/recipes/party/appetizers/party-appetizers-calculator/

5. Rattray D. How to estimate the amount of chicken you will need for each person [Internet]. The Spruce. 2018 [cited 2018 Feb 7]. Available from: https://www.thespruce.com/calculate-amount-of-chicken-or-turkey-to-buy-3057789

6. Scheidt DM, Daniel E. Composite index for aggregating nutrient density using food labels: Ratio of recommended to restricted food components. J Nutr Educ Behav. 2004;36(1):35–39.

7. Fulgoni VL, Keast DR, Drewnowski A. Development and validation of the Nutrient-Rich Foods Index: A tool to measure nutritional quality of foods. J Nutr. 2009 Aug 1;139(8):1549–1554.

8. Benjamini Y, Hochberg Y. Controlling the false discovery rate: A practical and powerful approach to multiple testing. J R Stat Soc Ser B Methodol. 1995;57(1):289–300.

1. William Benjamin Scott and Luna M. Scott Professor of Law; Professor, by courtesy, of Political Science, Stanford University; Senior Fellow, Stanford Institute for Economic and Policy Research

   * Corresponding author: [dho@law.stanford.edu](mailto:dho@law.stanford.edu) (DH) [↑](#footnote-ref-1)
2. Ph.D. Student, Harvard University. [↑](#footnote-ref-2)
3. Research Fellow, Stanford Law School. [↑](#footnote-ref-3)
4. J.D., Stanford Law School. [↑](#footnote-ref-4)
